# Reference-Free Validation of Short Read Data

**DOI:** 10.1371/journal.pone.0012681

**Published:** 2010-09-22

**Authors:** Jan Schröder, James Bailey, Thomas Conway, Justin Zobel

**Affiliations:** 1 Department of Computer Science and Software Engineering, The University of Melbourne, Parkville, Victoria, Australia; 2 NICTA Victoria Research Laboratory, Parkville, Victoria, Australia; Texas A&M University, United States of America

## Abstract

**Background:**

High-throughput DNA sequencing techniques offer the ability to rapidly and cheaply sequence material such as whole genomes. However, the short-read data produced by these techniques can be biased or compromised at several stages in the sequencing process; the sources and properties of some of these biases are not always known. Accurate assessment of bias is required for experimental quality control, genome assembly, and interpretation of coverage results. An additional challenge is that, for new genomes or material from an unidentified source, there may be no reference available against which the reads can be checked.

**Results:**

We propose analytical methods for identifying biases in a collection of short reads, without recourse to a reference. These, in conjunction with existing approaches, comprise a methodology that can be used to quantify the quality of a set of reads. Our methods involve use of three different measures: analysis of base calls; analysis of *k*-mers; and analysis of distributions of *k*-mers. We apply our methodology to wide range of short read data and show that, surprisingly, strong biases appear to be present. These include gross overrepresentation of some poly-base sequences, per-position biases towards some bases, and apparent preferences for some starting positions over others.

**Conclusions:**

The existence of biases in short read data is known, but they appear to be greater and more diverse than identified in previous literature. Statistical analysis of a set of short reads can help identify issues prior to assembly or resequencing, and should help guide chemical or statistical methods for bias rectification.

## Introduction

High-throughput or next-generation sequencing techniques are now cheap and widely available. Current machines, which produce short reads of up to around 500 bases, are emerging as a fundamental tool in biology and medicine. Short-read sequencing has replaced Sanger sequencing [1], [2] for applications involving long sequences such as chromosomes or whole genomes, and the availability of short-read sequencing has given rise to ambitious projects such as the *1000 genomes project* (www.1000genomes.org), which is using the technology to generate a detailed map of the genetic variation in humans. Applications of next-generation sequencing platforms involve identification of DNA protein interactions by ChIP-seq [3], transcriptome analysis with RNA-seq [4], or whole genome assembly [5], [6] of both known and novel organisms.

Short-read sequencing generates vast amounts of data, using the shotgun process. DNA molecules are amplified and then fragmented using techniques such as sonification or nebulisation. Portions of the DNA fragments are then sequenced by an iterative process involving fluorescence, digital photography, and image analysis, yielding *short reads* of some fixed length, from around 35 to 100 bases (for Illumina and SOLiD) and up to 500 for (Roche 454). Each of these steps, chemical and digital, may introduce biases and errors.

The short read data produced by sequencing machines is analysed using bioinformatics tools such as resequencers and assemblers. When using such tools, simplifying assumptions are commonly made: for example, that reads are evenly spread over the sequenced genome [7]–[9]; and that errors randomly occur within each read according to random substitutions. A richer assumption is that errors are more likely towards the end of the read, but are otherwise random as to base and location. However, as we demonstrate in this paper, such assumptions do not appear to apply to the data generated by one of the main current sequencing platforms; we find that the biases are more extreme and more complex than has previously been suspected.

Identification of bias in short-read data has been explored in other work, such as that of Dohm et al. [10] and Harismendy et al. [11]. Dohm et al. [10] focus on the Solexa 1G sequencing platform and measure aspects such as error rates, regional coverage, and biases towards particular sequences. The approach of Harismendy et al. [11] focuses on comparison of different sequencing technologies, analysing similarities and differences in their genome coverage. Ehrlich et al., Kircher et al., and Rougemont et al. independently published works in 2008–09 [12]–[14] that focus on the base-calling process as an alternative to the solution Bustard provided by Solexa (Illumina). The work of Kircher et al. [13] points out that systematic errors can be made by the standard software, arising from chemical and optical issues.

In contrast, the methodology that we present focuses on identification of biases in the short-read data itself. In particular, we explore detection of bias with respect to the distributions of bases and *k*-mers at distinct positions in the reads. An advantage of this approach is that it does not require the availability of any reference sequence, which also means it is less sensitive to errors caused by polymorphisms in the organism being investigated.

Our methodology consists of three related aspects: first, counting and comparing the frequency of bases at specific positions in the reads; second, counting and comparing the frequency of *k*-mers at specific positions in the reads; and third, evaluating and comparing the distribution of *k*-mers at specific positions in the reads.

We apply our methodology to short-read sequencing data generated by the Illumina platform for the 1000 Genomes Project. This project is notable due to its public profile and ambitious goals, and aims to produce data of high quality. Our analysis identifies strong, complex biases in the reads from this dataset. For example, the base A is significantly overrepresented at the start of reads, while *k*-mers such as poly-T are dramatically underrepresented; the *k*-mers in the middle few bases of the reads are distributed differently to the other *k*-mers. This analysis raises questions about the quality of the data and the ways in which the data might be correctly interpreted.

We do not attempt to use our analysis to identify the causes of the biases in the test cases we examine. Our primary aim is to develop general techniques that can be used for processing any short read data (though in particular data intended for assembly rather than peak analysis), and each experiment will have its own characteristics; that is, we propose a first stage of analysis that should be applied before any further processing is undertaken. These methods could be used to track down sources of bias, but could equally be used, say, to choose between data sets; for example, we have observed that different data sets – even different lanes from the same sequencing run – may have different characteristics. A further application is that they could be used in assembly or resequencing to augment other information such as quality scores.

We have developed a software package that allows the user to examine biases in a set of short-read data. In the following, we focus on whole genome sequencing data from the Illumina platform, but our methodology is generally applicable to other sequencing techniques and platforms, as is our *quarc* package (introduced in the next section).

The paper is structured as follows. We outline a mathematical model for describing the common assumptions that have been made about short-read sequencing data and present three simple, yet powerful, techniques to analyse and assess bias. We then apply our techniques to data from the 1000 Genomes Project, and demonstrate the presence of data quality issues.

## Materials and Methods

We present three simple, yet powerful, techniques for assessing a collection of short reads. They can be easily applied to any kind of read data and do not require any information about the organism. A reference sequence is not required, but is useful if available. We begin with an overview of each of our techniques.

### Technique 1: Analysing base calls

This simple measure is a count of how many times each specific base (A, C, G, T, or N) was called at given position in any read. Representing these base counts graphically can then provide hints about the general state of the data. If no bias is present, then one would expect equal counts at each position for a fixed base, and equal amounts within the two couples A&T and C&G, as there should be no bias between the forward and reverse strands in DNA. In general, the counts should directly reflect the C&G content of the sequenced material. Deviation from this expected outcome suggests possible biases in the data and where (in the reads) it may be present.

### Technique 2: Comparing occurrences of *k*-mers in the reads

Using a background model, not necessarily based on a reference sequence, we estimate the expected number of counts for given *k*-mers and then compare to their actual counts found at varying positions in the reads. In our experiments we consider *k*-mers of different lengths: 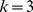, 4, 5, and 6. If no bias is present, one would expected relatively equal counts for a given *k*-mer at different read positions, and the count for each *k*-mer should reflect its content in the sequenced DNA. Again, deviation from this expected outcome suggests possible biases in the reads, allowing one to identify *k*-mers that are biased towards appearing at specific positions. If the background model is based on a reference sequence, then one can also identify which *k*-mers are generally under- or over-represented in the data. Note that technique 1 is a special case of technique 2 with 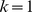.

### Technique 3: Analysing and comparing distributions of *k*-mers in the read set

In this method, at each particular read position we compute the distribution of the frequencies of all possible *k*-mers (of fixed length). We then assess bias by comparing distributions at different read positions, using the Kullback-Leibler divergence measure known from information theory. This yields an overall dissimilarity score between the two distributions of *k*-mers, with higher values indicating higher dissimilarity. We can then compare this score with an expected value obtained using a bootstrap approach; the method is explained in detail in the results section. If no bias is present, then we expect distributions from different read positions to have a low dissimilarity score that is close to the expected value.

### Modelling

Note that our methodology is generally applicable to any kind of sequencing data. The interpretation of the results however is specific to the kind of experiment the data was acquired through. The following assumptions apply to whole genome sequencing data only. When interpreting other experiments, such as ChIP-seq or RNA-seq, different hypotheses have to be formulated and applied accordingly.

Our methods can be used to test hypotheses about the data, which we formalise as follows. Let 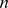 be the genome length, 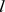 the read length, and 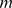 the number of reads. For a substring 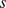, we define 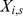 to be a random variable describing the number of reads that contain 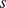 at position 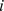. We can now model two standard assumptions.

#### Uniform distribution of reads in the genome

Under this assumption, each position in the genome is equally likely to be sampled with a read by the sequencing machine. This has the following consequence: if there are 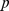 occurrences of a (short) *k*-mer 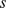 in the genome, every read has a probability 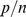 of starting with 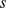. More precisely, let 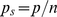, then the number of reads starting with 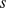 should follow the binomial distribution below. This forms a null “uniform sampling” hypothesis:
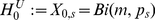
(1)


An implication of this assumption is that *k*-mers occurring in reads are equally likely to occur at any position within the reads. We can state an associated second null “position independence” hypothesis:
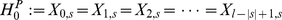
(2)In other words, the random variable 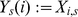 should be uncorrelated to the parameter 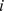. If the probability of a read starting with 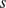 is 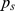, the same probability applies for all reads to have 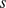 at the second position, the third, and so forth; for convenience we neglect the special cases at the start and end of the genome.

#### Random substitution error model

Under this assumption, the errors made by the sequencing machine do not occur uniformly across all positions in the reads, but, where they do occur, random substitution errors are assumed. We model this assumption as follows. Let 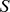 be a random variable representing a substitution at an error position in a read, then we can state a third null hypothesis:
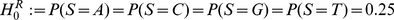
(3)where 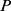 is the probability distribution for the substitution of a base.

### Datasets

We evaluate our techniques on a range of data sets coming from different sequencing platforms and organisms. The analysis includes a total of 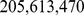 reads of various lengths, organisms, and sequencing platforms. The full list of read sets used can be found in Table S1. For the sake of consistency and comparability, we will present only one of the data sets in the main manuscript, a publicly available read set from the *1000 genomes project* (www.1000genomes.org).

The data 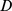 was generated by a recent edition of Illumina's Genome Analyzer II and is a union of the components 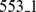, 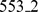, 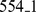, and 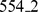, where each component is extracted from the NA10847 dataset (available at ftp://ftp-trace.ncbi.nih.gov/1000genomes/ftp/data/NA10847/sequence_read/). The data was generated with the *SBS v2* kit and processed by the *Pipeline v1.3* software package. This set is 6.6 GB of data in FASTQ format, consisting of approximately 52 million reads of length 51. Memory restrictions on our test hardware prevented us from using the entire set of data from the *NA10847* folder. Note that the volume of data we processed, and the size of the effects observed, ensures that results such as the differences in proportions of observed bases are statistically significant.

The reference for the human genome used in this paper is hg18 NCBI Build 36.1 from March 2006, available from genome.ucsc.edu/cgi-bin/hgTracks.

We have also successfully applied our analysis techniques to other datasets. Some results are included in the supplementary material and referenced in our discussions; we refer to this data as data sets *D1* to *D7*. The data presented in the main manuscript, 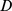, corresponds to *D5* in Table S1.

We filtered the data for artefacts that were present in the collection of reads, in order to exclude artificial biases. We filtered out poly-A fragments, which, users suggested, may occur frequently at the peripheral areas of the flow cells in the Genome Analyzer due to reflections. We also filtered out a sequencing primer starting with “GATTACAGGCATGAGC”, which we were able to identify after k-mer analysis of the data set.

### Software

Publicly available software for our analysis methods can be downloaded from www.genomics.csse.unimelb.edu.au/quarc. The package is called *quarc* (Quality Analysis and Read Control).

## Results and Discussion

We applied our three analysis techniques to our subset of the 1000 Genomes Project data, and used the model and null hypotheses proposed above to interpret the significance of our results.

### Analysing base call frequencies

**Figure 1:**
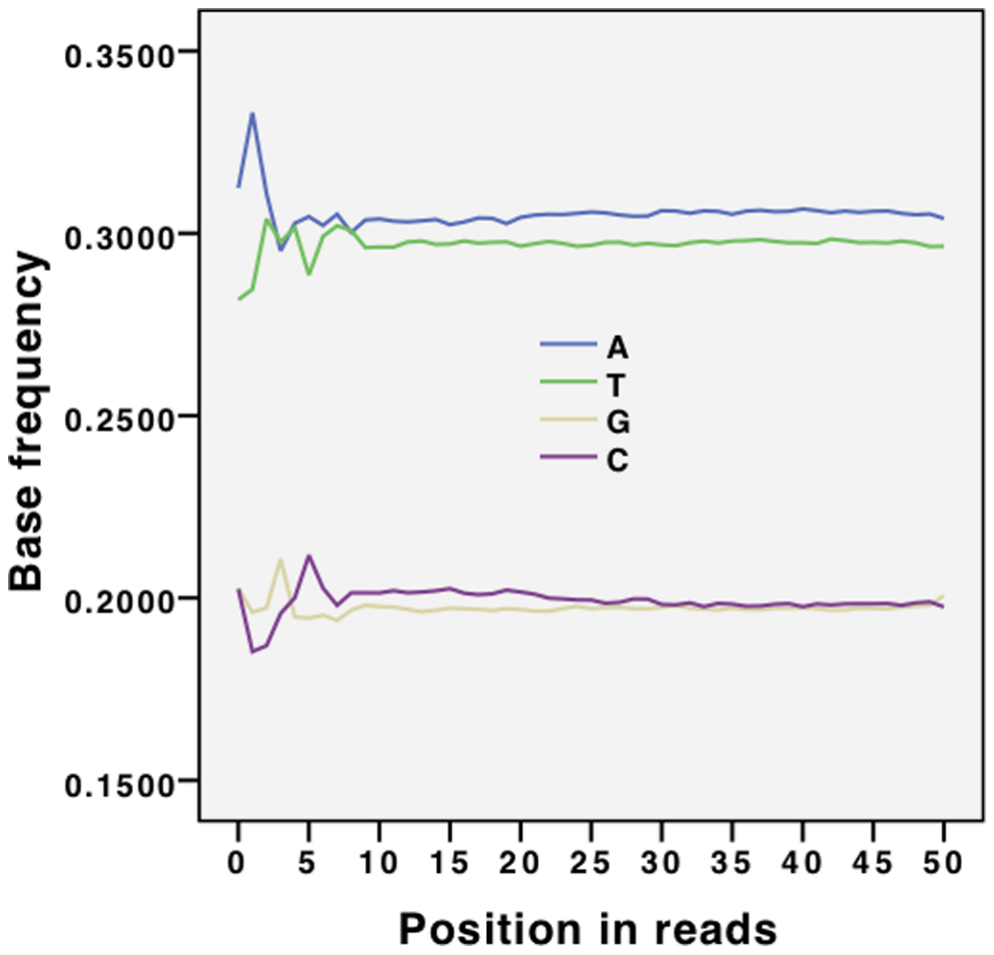
Basecalls for each position in the reads for dataset 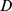 from the 1000 genomes project (see Section “Datasets”).

**Figure 2:**
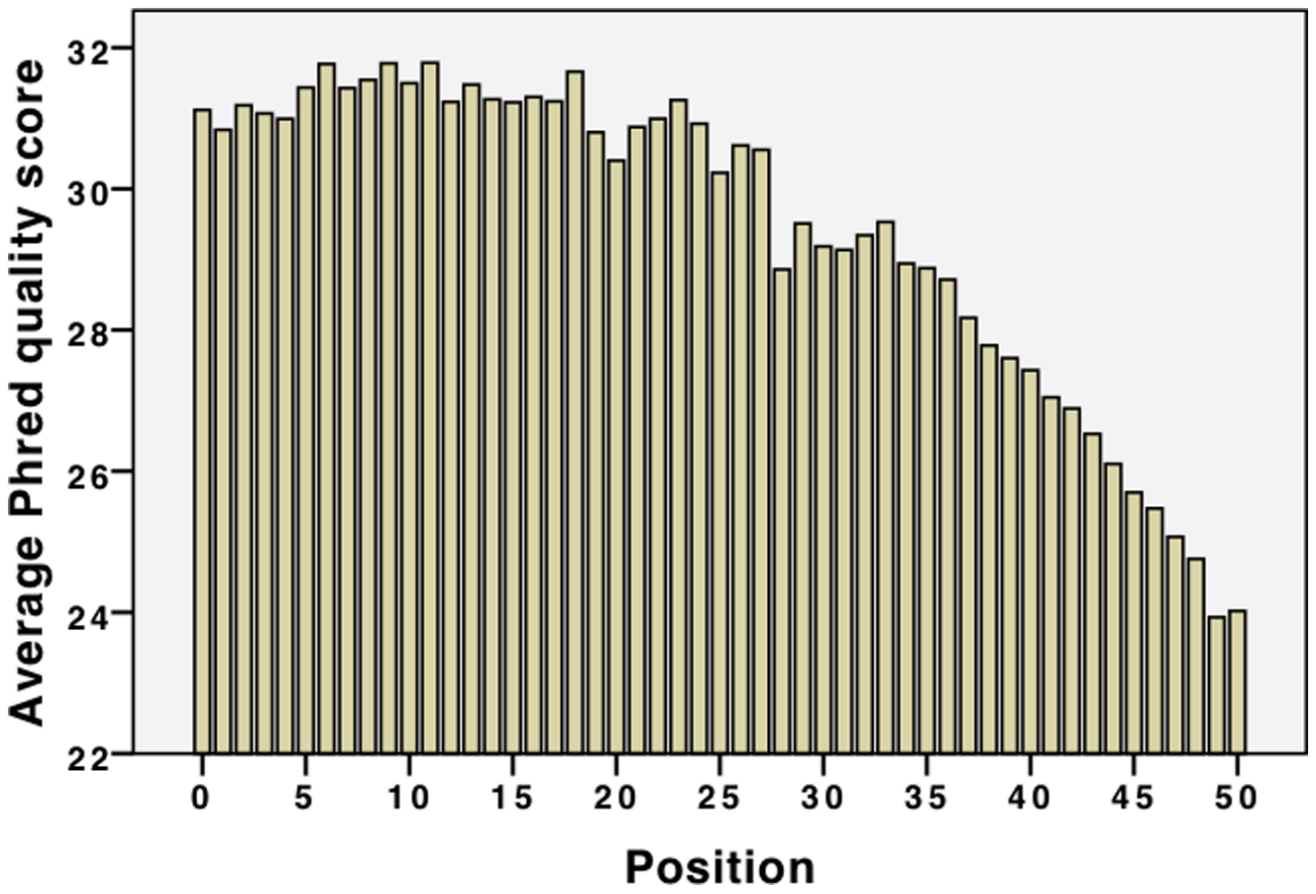
Average Phred quality scores for 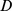. Bars represent the mean quality score for each position in a read (see [17] for details).

Our first approach to analysing the data was inspired by observations made by Dohm et al. [10] and the base-call analysis routines provided by Illumina (http://www.illumina.com/). Figure 1 shows a base-call graph for the dataset, and Figure 2 shows the accompanying quality values for these base calls. The following observations can immediately be made:

- A common assumption about short-read data is that base call frequencies should be independent of the position in the read. Figure 1, however, indicates that this assumption is only true from about base 10. The beginnings (bases 1 to 9) of the reads show great deviation from the expected behaviour.
- The deviant behaviour we observe across the initial bases cannot be attributed to the internal quality measures used by the sequencing machine. Figure 2 shows that there is no significant drop in quality score values across the first ten positions of the reads, indicating good reliability of the base frequencies at these positions.
- Figure 2 has strong similarity to a graph presented by Dohm et al. [10], showing a fall in quality scores toward the end of the reads. This result is also consistent with the observations made by Chaisson et al. in [15].
- The presence of major biases in the starting locations, or possibly the presence of sequencing primers left in the input data, could be responsible for the shape of the base call graph in Figure 1. We observed this behaviour (see Figures S1 and S2 as examples) in all the Genome Analyzer outputs we investigated.
- There is a noticeable enrichment of As over Ts and Cs over Gs in the base call frequencies. This is true for all of the Illumina Genome Analyzer II read sets investigated in the supplementary data as well (except one that is not representative of a regular sequencing run). Such differences in complementary bases could be explained with strand sampling biases. However, a strand bias can't explain the consistent preference for one base over the other. Poly-A fragments (mentioned in the data set section) that have not been removed from the data could play a role in oversampling of As, but there is no analogous phenomenon that would explain the difference between Cs and Gs. An amplification and sampling bias may be the cause for the observation.
- Note that the same observation can be made for the 454 data regarding AT, but the roles are switched for CG (see Figure S8).

The base calls are exhibiting this behaviour, even after our filtering of the dataset for primers and other artefacts, as described in Section “Datasets”. We discovered this kind of pattern as a common characteristic for base calls coming from Illumina sequencers; see the supplementary data for further analysis.

The data sets *D1* and *D4* in the supplementary data have been mapped to their reference genomes to ensure only valid reads. Furthermore, the reads of D4* have been quality filtered, so that we only retained reads in which all bases have a high quality score. Our aim is to develop methods that do not require a reference, but it is plausible to hypothesise that the biases are due to poor reads; this mapping and filtering eliminates poor reads as an explanation for the biases.

Analysis of *D3* (another *1000 genomes* dataset) revealed another striking anomaly in base call frequencies, shown in Figure S3, with base frequency varying wildly with position and significant falloff in calling of A towards the end of the read. We believe it is important to be aware of such characteristics before undertaking genome assembly.

Other sequencing platforms show different error characteristics, which then shows in the base call frequencies. Data set *D7* was created with Roche's pyrosequencing technology 454; its base call progression can be reviewed in Figure S8. There is a noticeable increase of A nucleotides towards the end of reads, whereas the occurrences of Cs decline. Note however, that this read set is composed out of various read lengths, and the observed behaviour could be explained by sampling biases as well as error biases.

Being aware of these characteristics of the input data can help to better interpret them, thus demonstrating the value of applying simple statistics of this kind of data before trying to make use of it. For instance, base call progressions at the end of reads, such as those in the data sets *D1*, *D4*, *D5* (*D*), and *D7* (see Table S1), suggest that users should make use of trimming techniques like that presented by Qu et al. [16].

### Analysing occurrences of *k*-mers

Our second technique examines the frequency of *k*-mer occurrences against a background model. Having observed anomalies occurring at the start of reads identified in Section “Analysing base call frequencies”, we generalised the concept and compared the frequencies of *k*-mers for varying 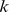 at the read start against their overall frequencies in the reference sequence or, if not available, with a background model derived from the rest of the reads. As was formalised in “Modelling” section, we would expect that each position in the genome is equally likely to be sampled by a read and thus the *k*-mers at a start of the read should follow the distribution of the *k*-mers in the reference or background (methylation issues excluded). However, we found large discrepancies from this assumption in our analysis, with *k*-mers at the start of a read being under- or over-represented with respect to the background model by orders of magnitude.

In more detail: we obtain the probability distribution for all *k*-mers from a reference genome as a background model. No reference is ideal; some genomes are CG-rich to a greater extent than others, for example, while others have such a high proportion of coding region (such as Drosophila) that other forms of non-randomness may be observed. However, a reference from a similar organism should have reasonably similar statistical properties. As noted below, the pool of reads themselves can be used as a background model.

According to our null hypothesis 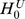, the distribution of any *k*-mer should be binomial 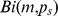. Since 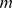 is large and 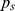 is small for this kind of data, we calculate 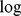 values of the distribution using the Sterling approximation (that is, we approximate the factorials for large numbers as 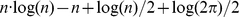). We can then obtain p-values by approximating the cumulative probabilities for the given binomial distribution taking the observed values; for values far from the distribution's mean we estimate the tail by 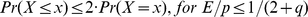, 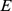 the mean and 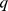 the binomial probability. The p-values obtained for certain *k*-mers show highly significant differences from the expected values at the 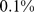 confidence level. This provides strong evidence to reject the 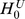 hypothesis.

**Figure 3:**
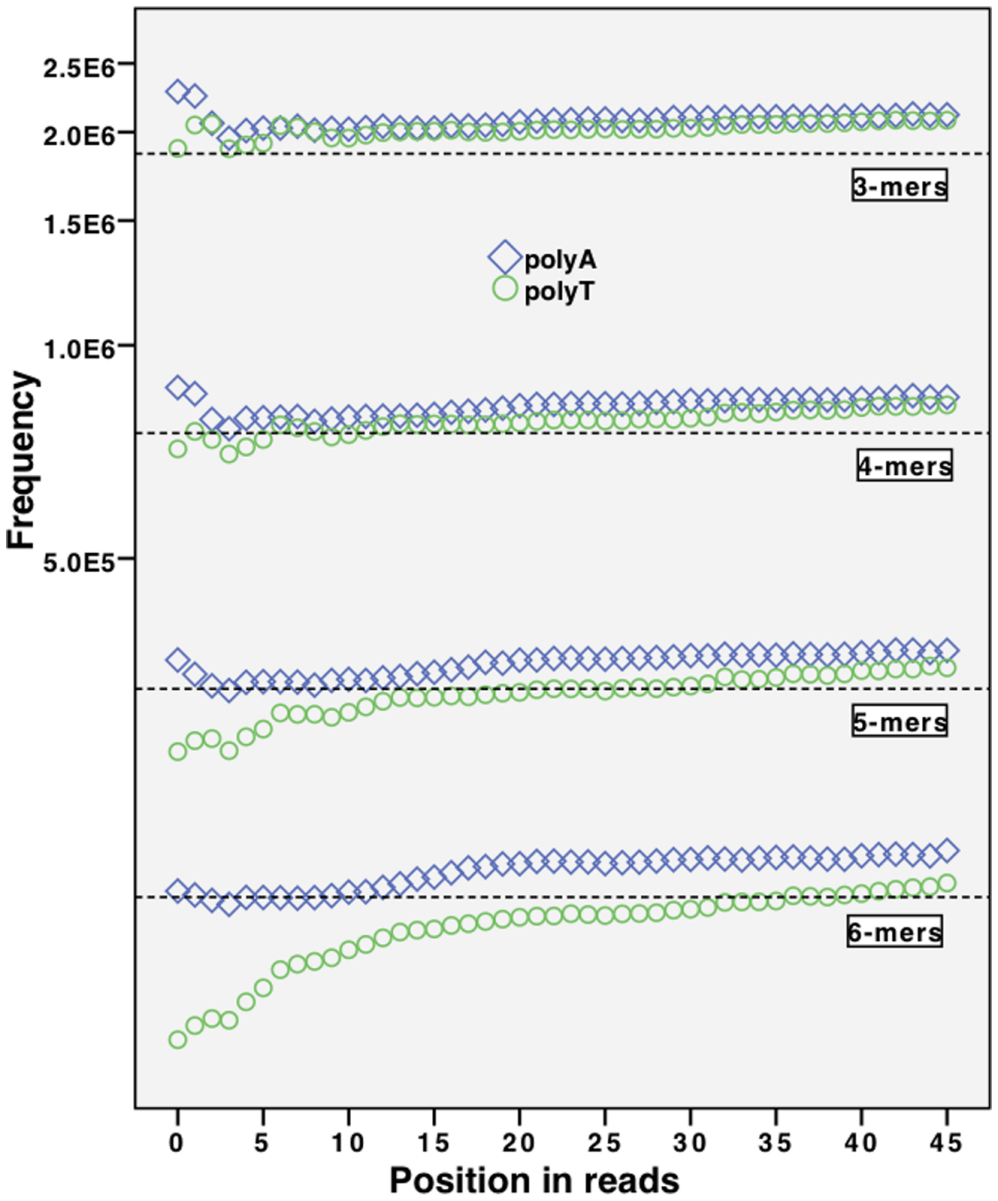
Occurrences of poly-A and poly-T sequences of different length depending on position in the read and their expected values for read set 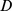. y-axis shows the total number of occurrences (log scale). Dotted lines represent the expected occurrences for the respective *k*-mer lengths. Poly-A sequences displayed as 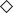, poly-T as 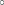.

**Figure 4:**
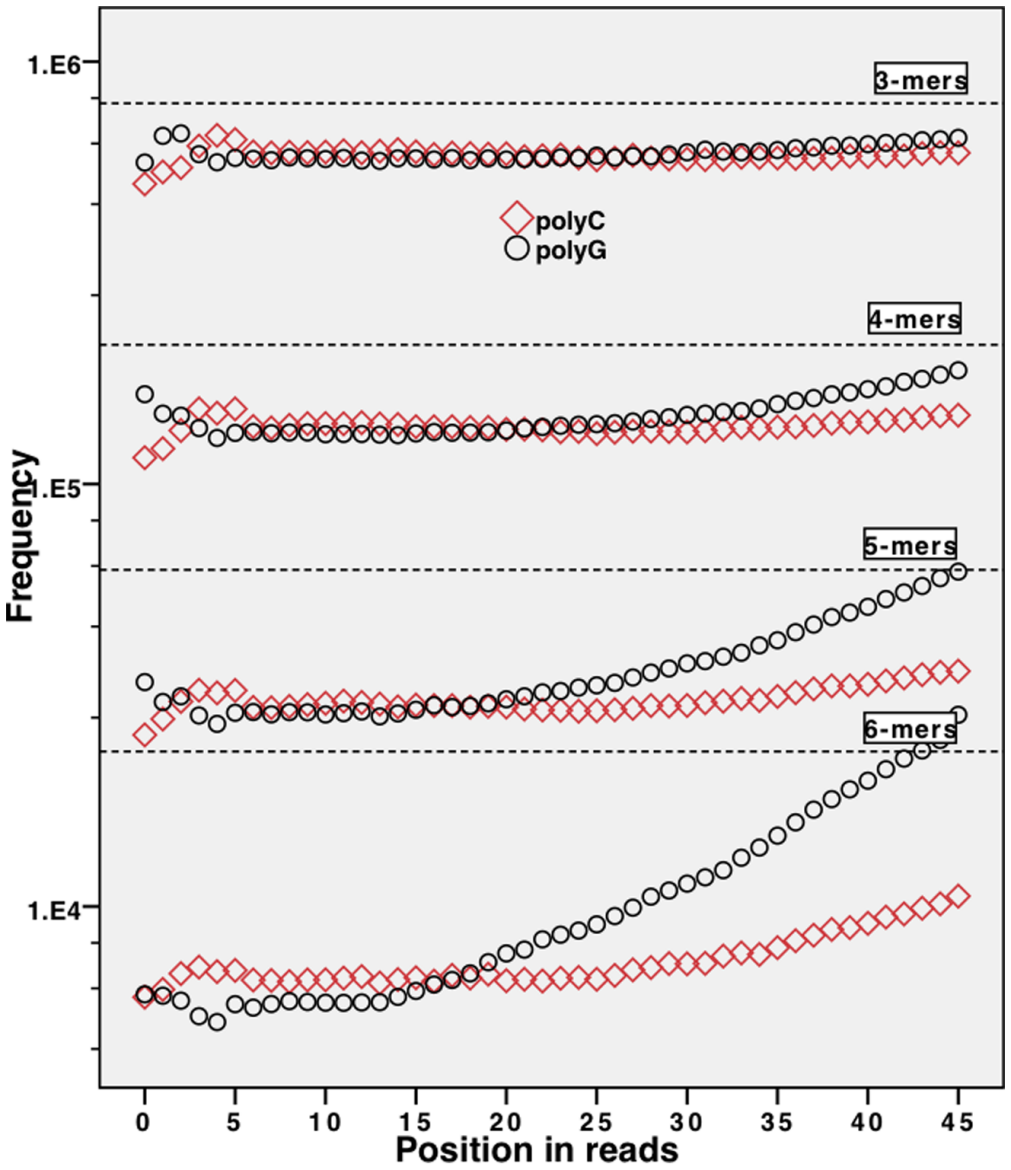
Occurrences of poly-C and poly-G sequences of different length depending on position in the read and their expected values for read set 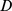. y-axis shows the total number of occurrences (log scale). Dotted lines represent the expected occurrences for the respective *k*-mer lengths. Poly-C sequences displayed as 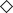, poly-G as 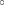.

We identified some of these anomalies as PCR primers contained in the reads (as for example in the NA06985 read set presented in the supplementary data). Other anomalous *k*-mers could not be explained as easily and, more curiously, showed consistently unusual behaviour across completely unrelated datasets. The polynucleotide sequences are a notable example. Our experiments with several datasets showed an under-representation of poly-C, poly-G, and poly-T sequences at the read start – and in general compared to the reference genome – whereas the reverse complement of poly-T, namely poly-A, occurred well in the expected range. The calculated p-values for poly-C, poly-G, and poly-T *6-mers* are all significant at the 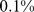 significance level. We also analysed where such polynucleotide sequences occurred in the reads. Figures 3 and 4 show the frequency for *k*-mers of repeated nucleotides at each position in a read, with the dotted line showing their expected frequency based on the reference genome. Note the interesting and significant difference of representation of identical strings in complementary form (poly-A versus poly-T and poly-C versus poly-G).

The frequencies of these anomalous *k*-mers show significant correlation with their position of occurrence in a read. Squared correlation coefficients and their p-values are shown in Table 1. The statistics were generated using the PASW statistics 17.0 software and cubic interpolation was found to provide the best fit when finding the correlation between read position and frequency for a given *k*-mer. We summarise our observations as follows:

- The highly significant p-values in Table 1 suggest that frequencies of certain *k*-mers are not independent of their position in a read. We thus reject the null hypothesis 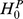 and hence also reject 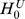.
- All the polynucleotide sequences show increased frequency towards the end of reads.
- Unusual behaviour is found at the start of reads, where the selected *k*-mers have unusually high or low frequencies (see Figure 3). We found this kind of behaviour repeated across different datasets.
- The majority of *k*-mers (that is, those other than the polynucleotide strings) don't show this kind of behaviour and their frequency distributions are consistent with the null hypothesis.
- Comparing our results from Figure 4 with Figure 1 shows that there is a dramatic increase of poly-G 6-mers at the end of reads, even though the count of base calls for single G bases remains stable towards the end of reads.
- Polynucleotide sequences consisting of C or G are significantly less represented in the reads compared to the reference genome. This stands in contrast to the observations made by Dohm et al. [11], who detected an enriched representation with higher CG content. This bias however, was observed in Solexa 1G sequencers. Other unpublished experiments we have undertaken show that this bias is not prevalent for later editions of the Illumina sequencing platform.
- Experiments show that the higher occurrences of poly-A, poly-T, poly-C, or poly-G sequences at the end of reads is not a reflection of the material being sequenced but is due to a systematic introduction of error, turning quasi poly-nucleotide sequences into actual ones by minor changes. We verified this by mapping the reads to a reference genome and identifying erroneous bases in uniquely mapping reads: The majority of poly-G sequences in the read data were seen to occur due to systematic sequencing errors, violating the assumption 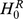. This contradicts our error models, because errors are not context free.

**Table 1:** Cubic correlation coefficients for polynucleotide sequences of different length for the read set 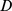.

	polyA	polyC	polyG	polyT
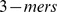				
Correlation 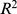	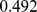	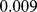 *	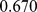	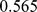 *
p-value	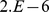	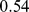 *	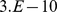	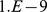 *
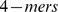				
Correlation 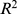	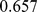	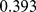	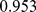	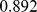
p-value	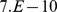	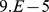	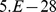	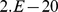
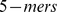				
Correlation 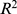	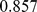	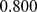	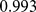	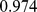
p-value	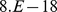	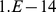	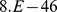	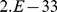
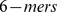				
Correlation 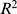	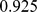	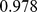	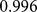	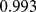
p-value	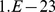	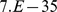	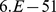	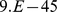

*Values for linear correlation (better fit).

Although we show results here using a reference sequence, this method, as the others, can be applied without a reference. The reference has been used to validate our methodology, not as an essential step to obtain results. Note that, in absence of a reference, the pool of reads themselves can be used to obtain a background model, and that the positional analysis of *k*-mer frequencies is entirely independent of a reference in the first place.

As was the case for the first method we presented, this second simple statistic can provide useful quality information on a set of reads, and help guide later computational or chemical analysis. For example, our results illustrate that the biases are probably due to the chemical processing rather than the sample preparation, as the data sets were prepared in different laboratories. Although the choice of data to use as a background model may lead to apparent biases, our results here suggest there are other more significant causes. This is confirmed by the Kullback-Leibler analysis, as we next explain.

### Distribution analysis

Our third proposed analysis assesses the data in more depth. Given the observation in Figure 3 and 4 and the uneven distribution over the read positions of distinct sequences, we analyse the overall *k*-mer distributions for each position in a read. We then compare distributions using the Kullback-Leibler (KL) divergence to get an intuition of how different the distributions are.

Given two probability distributions 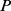 and 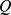, the KL divergence of a set of *k*-mers 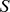 is:
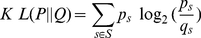
(4)where 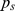, 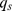 denote the probability of 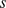 under 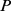 or 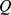 respectively. Intuitively, this function measures how different two distributions are, a higher value implying higher divergency. In information theory, the KL divergence measures the average amount of bits wasted per symbol when using distribution 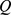 for encoding when symbols are in fact distributed according to 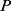.

**Figure 5:**
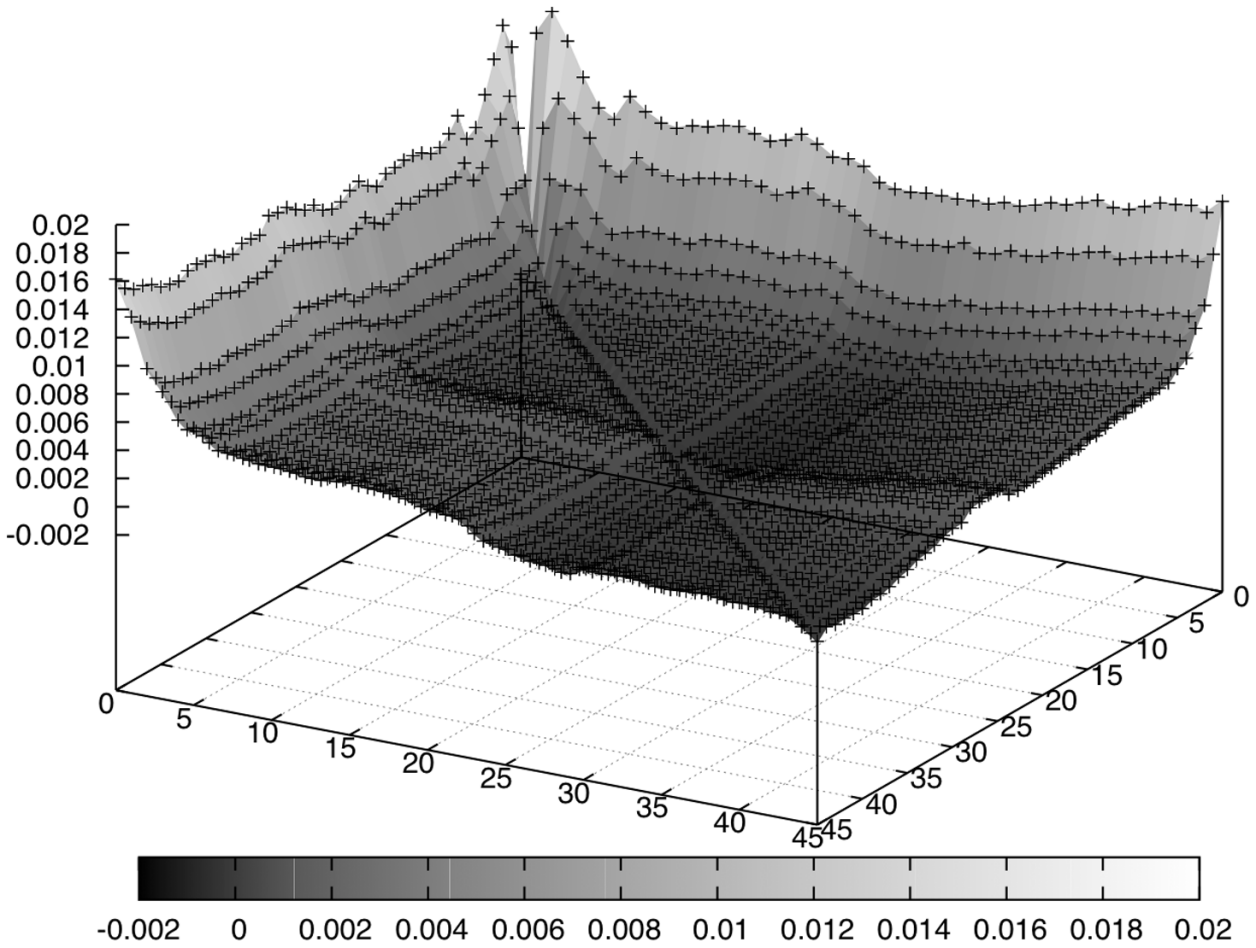
3d plot of the KL matrix for 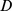. Data points correspond to 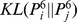, 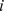 shown on the x-axis, 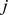 on the y-axis.

Let 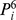 be the distribution for all *6-mers* at position 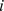. We then compute the KL divergence between each possible pair of positions: 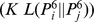, 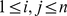, where 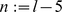 is the number of *6-mers* in a read, with 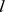 the read length. Figure 5 shows a graphical representation of the divergence profile.

- Divergence is high when comparing the first position's distribution against any other. This might imply biases in the starting positions of reads and thus the existence of biased *6-mers* in the first bases.
- Divergence is high when comparing the first with the last position's distribution. This observation is valid across all analysed datasets – and expected as explained later.
- The main area of the graph contains small divergence measures of around 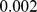. This ‘plain’ of small divergences seems to confirm the claim made earlier in the “Modelling” section, that for the majority of *6-mers*, their occurrences are consistent with the null hypothesis 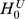, thus also implying 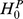.
- There is a small but significant “bump” in the divergence when comparing any positions with those around 25 to 30. This can be seen as lighter coloured stripes crossing horizontally and vertically through the middle of Figure 5. We believe it to be caused by artefacts left in the data. Factors such as primers occur in a particular reading frame and can cause biases in the distributions at particular loci of the reads.
- Besides the obvious extreme values for divergence stated above, there is a more subtle but clearly visible decline in divergence from the first few positions towards the end of the reads, in general, divergence is higher for early position's distributions than for later ones. This also coincides with the Figure for the bootstrap experiment presented shortly.
- Across several datasets, the general shape of the graph representing the KL divergences was similar: maximal divergence occurred for the first position and was high compared to any other position. See Figures S9 to S16 for illustration.

To assess the observed divergences, we use a form of bootstrap. We use the *maq* (maq.sourceforge.net) simulation tool to generate multiple synthetic read sets from the human genome, of the same size as our natural data set, using the assumptions underlying 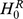. We first train the simulator using the quality scores from our test data and then generate 100 different sets of reads (each with around 52 million reads) from the reference genome based on the adopted quality scores. For each read set, the KL divergence values are computed and then an average is taken over all read sets. With this approach we simulate the expected value and the variance of the KL divergence values under the uniform sampling null hypothesis 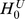.

**Figure 6:**
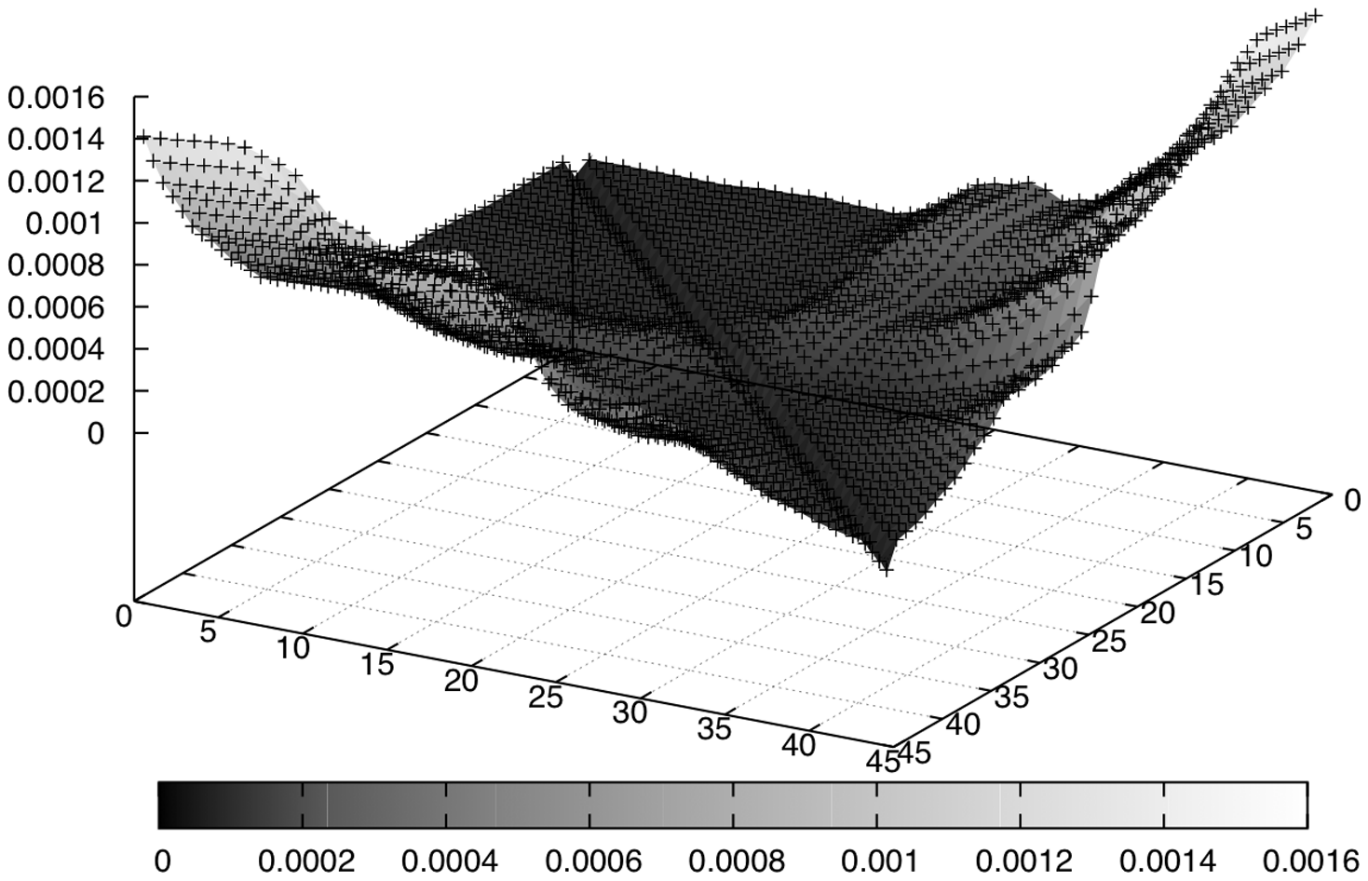
3d plot of the mean KL values for the bootstrap approach (simulated data). Data points correspond to 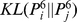, 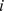 shown on the x-axis, 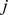 on the y-axis. Note that the scale is not the same as used in Figure 5.

The central limit theorem then implies that the divergence measure should be normally distributed around the distribution's mean. Mean and variance are derived directly from the simulated distribution. Figure 6 shows the expected values for the KL divergence under the null hypothesis; as can be seen, these are much smaller than the values observed for the real data. (Note that the vertical axes are on different scales.) Table 2 shows further results; instead of p-values we provide effect size in distance from the mean as multiples of the standard deviation of the respective distribution.

**Table 2:** Statistics for the bootstrap approach and comparison with the read data.

		Statistic
Average standard deviation from mean		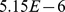
95% confidence interval for	(lower bound)	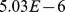
mean standard deviation	(upper bound)	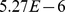
Avg. distance of observed values from expected value*		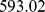
Avg. effect size for 1st position distr. from expected values*		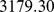

*In standard deviations.

The general ‘stingray’ shape of the graph in Figure 6 is initially surprising, but is a direct consequence of the error model adapted by the simulation tool. Recall that we trained the simulator with the quality scores of the dataset (see Figure 2). The higher probabilities for errors at the end of reads leads to a higher diversity of the 6-mer distributions and such to the observed graph; note that the distribution of 6-mers in the human genome is biased, so that introducing errors using random substitutions tends to make the distribution become more even towards the end of the reads. Note further that increasing error rates under the assumption of 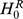 makes the *k*-mer distributions converge towards a uniform distribution, since eventually every position in a read is replaced by an error with equal probability for each base. Even though the error model adopted here does not capture all of the errors in real data, it does reflect the notion of increasing error rate towards the ends of reads. Thus, we expect higher divergence between early and late distributions, because the errors corrupt the pattern of 6-mers observed.

The divergence measure can be applied for any 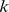. Large 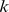 will result in low sampling of each *k*-mer and thus lower the statistical significance. Also, some *k*-mers might never be sampled at some positions in the read, whilst being contained at other positions, resulting in difficulties in calculating the KL divergence. Smaller 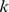 results in little specificity of the *k*-mers to a region in the genome, and thus reduces the power of the method to discover regional biases. The choice for length six for this analysis however was arbitrary.

### Discussion

DNA sequencing is a complex process combining several stages of preparation, chemistry, and computational analysis. Biases for distinct *k*-mers or fragment lengths can be introduced at many points during this process: PCR can favour certain *k*-mers, for example, as can DNA fragmentation. The chemistry used inside the sequencing hardware and the interpretation of the optical reactions is sensitive to interferences of many kinds, such as light and temperature. Our observations imply that some unexpected, complex biases are present in data from the 1000 genomes project, and that these may affect how the data is interpreted.

Table 1 shows that some sequences' occurrences are highly correlated with the position in the reads, contradicting assumptions of how reads are obtained from a DNA sequence. This correlation could be due to the preparation steps of the sequencing library or biases in the sequencing step, or it could be a systematic error in the interpretation of the reaction in the sequencer. These kinds of errors are noted by Harismendy et al. [11] and in more detail by Kircher et al. [13]. For example, this includes the tendency of G to be confused with T, and also a general T accumulation along the reads. The latter was observed in some but not all of our experiments (see Figures S1 to S8).

On the other hand, it could be a correct image of the data and caused by biases in the starting positions of the reads. This is confirmed by results presented in the supplementary data: the data set D4* was mapped to the reference and quality filtered to ensure only high quality reads that stem from the actual organism with high probability. Figures S7 and S15 show the graphs for base call frequencies and the KL divergence measure. The results show no improvement in the observed biases.

Biases in the starting positions of reads become apparent when looking at the other analyses. Figure 5, representing the Kullback-Leibler divergence of different positions' distributions, shows that the starting positions of reads do not coincide with the general null hypothesis or with the general shape of distributions at other positions. One has to be careful about interpreting the possible biases, because adaptor sequences or any fragments that appear in a distinct reading frame in a read may lead to this observation. Quality filtering however suggests that this is not the case. Figure S15 looks slightly improved over Figure S11, with smaller divergence for late positions in reads. The divergence at the start of reads however remains present. We thus believe that the underlying issues are not simple sequencing errors or fragments left in the data, but rather systematic biases in site selection in the read generation process.

However, we did filter the data (recall the “Datasets” section) to get a clearer image of the state and the observation persisted. We also note that, even if primers or other artefacts were somehow left in the data, the shape of the graph should look different if the reads were unbiased in their starting positions: A primer that occurs at the start of reads massively biases the distribution for the first position – but it also does the same for the second, third, and so on, for a large number of positions, as adaptor sequences or primers are typically long. That is, if the graph's shape is due to this kind of phenomena, the high divergence should stretch further into the reads.

The same argument applies to Figure 1, where the unusual *k*-mers should certainly exceed 10 bp. Thus use of our techniques can give insight into these biases in read starting positions: analysing the over- and under-represented sequences at the starts of reads by calculating p-values as described in Section “Analysing occurrences of k-mers” might indicate favoured and avoided positions in a genome on a sequence level.

A criticism of the analysis in Section “Distribution analysis” could be that the comparison to a reference mightn't be fair: the actual read data could be biased due to initial sample preparation from the genome and the sequence might simply be different from the reference. However, these issues should not affect the overall distributions significantly and, in particular, should not affect the general shape of the graph at all, which this is determined by our assumptions only and not by the sequence the reads stem from. Recall that we do not compare the read data with the reference genome in this step, but distributions along the reads themselves. Using the reference for the bootstrap however ensures maintenance of the same genome complexity and coverage ratio as in the test data.

Practical experience demonstrates that short read data is feasible for the common tasks of re-sequencing or assembly [5], [6]. Yet we need to be aware of possible biases and try to understand the underlying characteristics of short-read data better to make the most of the information contained in it, and doing so may aid in construction of longer contigs with greater coverage, or in accurate determination of genome regions involved in gene expression.

Our statistical tests have practical implications for a wide variety of biological investigations. For example,

- Combining the results from base call and distribution analyses, reads can be trimmed in a guided manner: The trimming points can be chosen in a manner to maintain as much sequencing material as possible while minimising errors. The results of Qu et al. [16] show that a significant volume of errors can be omitted this way. This increases the mappability of the data in case of resequencing, RNA-seq, and so on.
- Based on the same observations, a guided kmer selection for kmer-based assembly algorithms can leverage performance for de novo assembly applications. Avoiding read regions of the data set that contain high error rates and bias, will benefit the assembly quality and performance, because avoiding errors makes assembly of the short read data easier, and it drastically reduces the memory consumption of assembly tools – one of the main problems for sequencing larger genomes.
- With the results from distribution and *k-mer* analyses, a more accurate coverage estimation for quantitative analysis such as ChIP-seq or RNA-seq can be achieved. The statistical tests that are used for this kind of experiment are highly sensitive, and rely on accurate estimations of the gene (or RNA) coverage. Evaluating sampling biases and normalising for them could greatly improve the accuracy of gene expression studies with NGS data.

As new uses of short-read data continue to appear, we expect that precise knowledge of the data's statistical properties will continue to be of importance.

### Conclusions

We have presented strong evidence that the common assumptions made about short-read sequencing data are inaccurate. There seem to persist chemical or mechanical biases in the process that lead to surprising biases, such as overrepresentation of some k-mers in the middle of reads. We have to be aware of these biases when working with the data. When analysing methylation or expression characteristics, for example, biases in coverage can lead to mis-interpreted results if ignored. In terms of sequence assembly a notion of locality of *k*-mers stemming from particular positions could help improve the quality.

We presented new, simple tests and demonstrated that they provide insight into the sequencing data's state. The results pose questions about the quality and characteristics of high throughput sequencing data, and that of the 1000 Genomes Project in particular. We therefore recommend application of our techniques to maximise the use of information contained in the data and to better understand experimental results.

The base call analysis is easiest to apply and can give a good first impression of the data's state. A smooth graph will indicate the desired characteristics of the read data, while fragmented patterns indicate a problem. Counting occurrences of *k*-mers can help identifying such artefacts and filter them, but also aid understanding about more complex characteristics of the sequencing data such as positional biases. Applying the Kullback-Leibler measure helps to assess the state of the read data in more depth; a ‘smooth’ set of divergence values implies a homogenous read set, while any conspicuous patterns in the divergences identify biases and can help to direct further chemical and computational analysis.

## Supporting Information

Table S1 (0.07 MB PDF)

Figure S1 Basecalls for the read set D2 (NA06895) from the 1000 Genomes Project. X-axis showing the position in the read, y-axis the relative base frequency.
(0.63 MB TIF)

Figure S2 Basecalls for the read set D6 (NA12272) from the 1000 Genomes Project. X-axis showing the position in the read, y-axis the relative base frequency.
(0.95 MB TIF)

Figure S3 Basecalls for the read set D3 (NA11829) from the 1000 Genomes Project. X-axis showing the position in the read, y-axis the relative base frequency.
(0.72 MB TIF)

Figure S4 Basecalls for the read set D4 (NA12155) from the 1000 Genomes Project. X-axis showing the position in the read, y-axis the relative base frequency.
(1.63 MB TIF)

Figure S5 Basecalls for the read set D1 (SRX005986) from NCBI's Sequence Read Archive. X-axis showing the position in the read, y-axis the relative base frequency.
(1.78 MB TIF)

Figure S6 Basecalls for the read set D5 (NA10847) from the 1000 Genomes Project. X-axis showing the position in the read, y-axis the relative base frequency.
(2.87 MB TIF)

Figure S7 Basecalls for the read set D4* (NA12155) from the 1000 Genomes Project. X-axis showing the position in the read, y-axis the relative base frequency.
(1.57 MB TIF)

Figure S8 Basecalls for the read set D7 (SRX017210) from NCBI's Sequence Read Archive. X-axis showing the position in the read, y-axis the relative base frequency. Note that the graph is cut off at position 361, because only a very small number of reads exceeds this read length.
(3.46 MB TIF)

Figure S9 Kullback-Leiber divergence for the read set D1 (SRX005986) from NCBI's Short Read Archive. Data point represent KL(P_i_∥P_j_), x-axis indexing the first distribtion, y-axis the latter. P_i_ corresponds to the distribution of 6-mers at the ith position in a read. Note that the graph has been trimmed of the last position's distribution because of the high error rates.
(6.32 MB TIF)

Figure S10 Kullback-Leiber divergence for the read set D2 (NA06985) from the 1000 Genomes Project. Data point represent KL(P_i_∥P_j_), x-axis indexing the first distribtion, y-axis the latter. P_i_ corresponds to the distribution of 6-mers at the ith position in a read.
(3.12 MB TIF)

Figure S11 Kullback-Leiber divergence for the read set D4 (NA12155) from the 1000 Genomes Project. Data point represent KL(P_i_∥P_j_), x-axis indexing the first distribtion, y-axis the latter. P_i_ corresponds to the distribution of 6-mers at the ith position in a read. Note that the graph has been trimmed of the last position's distribution because of the high error rates.
(6.32 MB TIF)

Figure S12 Kullback-Leiber divergence for the read set D5 (NA10847) from the 1000 Genomes Project. Data point represent KL(P_i_∥P_j_), x-axis indexing the first distribtion, y-axis the latter. P_i_ corresponds to the distribution of 6-mers at the ith position in a read.
(3.52 MB TIF)

Figure S13 Kullback-Leiber divergence for the read set D6 (NA12272) from the 1000 Genomes Project. Data point represent KL(P_i_∥P_j_), x-axis indexing the first distribtion, y-axis the latter. P_i_ corresponds to the distribution of 6-mers at the ith position in a read.
(3.44 MB TIF)

Figure S14 Kullback-Leiber divergence for a chip-seq data set. Data point represent KL(P_i_∥P_j_), x-axis indexing the first distribtion, y-axis the latter. P_i_ corresponds to the distribution of 6-mers at the ith position in a read.
(2.91 MB TIF)

Figure S15 Kullback-Leiber divergence for the read set D4* (NA12155) from the 1000 Genomes Project. Data point represent KL(P_i_∥P_j_), x-axis indexing the first distribtion, y-axis the latter. P_i_ corresponds to the distribution of 6-mers at the ith position in a read.
(6.32 MB TIF)

Figure S16 Kullback-Leiber divergence for the read set D7 (SRX017210) from NCBI's Short Read Archive. Data point represent KL(P_i_∥P_j_), x-axis indexing the first distribtion, y-axis the latter. P_i_ corresponds to the distribution of 6-mers at the ith position in a read. Note that the graph is only displayed up to postition 250, since the very low number of reads exceeding this read length makes comparison of distributions difficult and little meaningful.
(6.32 MB TIF)
