# Supplementary material for: Reference-Free Validation of Short Read Data
Source: PLoS One. 2010 Sep 22;5(9):e12681. doi: 10.1371/journal.pone.0012681 (PMC2943903; doi:10.1371/journal.pone.0012681)
Supplement: Table S1 — (0.07 MB PDF) [file pone.0012681.s001.pdf]

Table S1: We analysed the following data sets of short Illumina and Roche 454 reads:

Identifier	Experiment name	Accession Number	Organism	Instrument model	Read length	Number of reads
D1	SRX005986 (NCBI SRA)	SRR018090	Drosophola melanogaster	Illumina Genome Analyzer II	45	8505994
D2	NA06985 (1000 Genomes Project)	ERR001014, ERR001015, ERR002406, ERR002407	Homo sapiens	Solexa 1G Genome Analyzer	35-37	22414082
D3	NA11829 (1000 Genomes Project)	SRR003488	Homo sapiens	Illumina Genome Analyzer II Solexa-5374	36	11442213
D4,D4*	NA12155 (1000 Genomes Project)	SRR00312[1-6]	Homo sapiens	Illumina Genome Analyzer II Solexa-6388	51	87872470
D5	NA10847 (1000 Genomes Project)	ERR000553, ERR000554	Homo sapiens	Illumina Genome Analyzer II	51	51116704
D6	NA12272 (1000 Genomes Project)	SRR015432, SRR015424	Homo sapiens	Illumina Genome Analyzer II	51	23739801
D7	SRX017210 (NCBI SRA)	SRR036930	C. botulinum	Roche LS454	35-402	522206

Data preparation:

All data sets have been filtered for poly A reads that occur in Illumina read sets. Although poly A regions may occur in the sequenced genome, these reads are likely to be misread due to reflections at the peripherie of the flow cells.

Data sets *D1* and *D4* have been mapped to their reference genome to guarantee valid reads without any adaptors or primers left in the data. The reads have been mapped with *SOAP2* against their references *Release 5* for Drosophila and *hg19* for human respectively (allowing up to two mismatches and taking only uniquely mapping reads):

- For *D1* 6457223 reads mapped uniquely to the reference (75.9%).
- For *D4* 66521869 reads mapped uniquely to the reference (75.7%).

Furthermore, we applied quality filtering to the *D4** read set:

- Only reads with a phred quality score over 10 (corresponding to a confidence of over 90%) in every base have been taken into account. Note that the mapping was applied to *D4** as well. 27235724 passed the filtering stages (31%).
