# Supplementary figures and images for: Reference-Free Validation of Short Read Data
Source: PLoS One. 2010 Sep 22;5(9):e12681. doi: 10.1371/journal.pone.0012681 (PMC2943903; doi:10.1371/journal.pone.0012681)

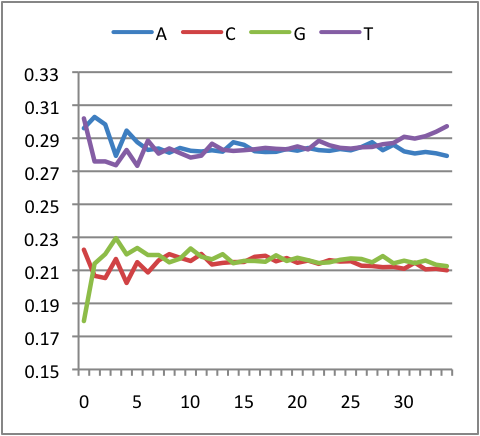

Supplement: Figure S1 — Basecalls for the read set D2 (NA06895) from the 1000 Genomes Project. X-axis showing the position in the read, y-axis the relative base frequency. (0.63 MB TIF) [file pone.0012681.s002.tif]

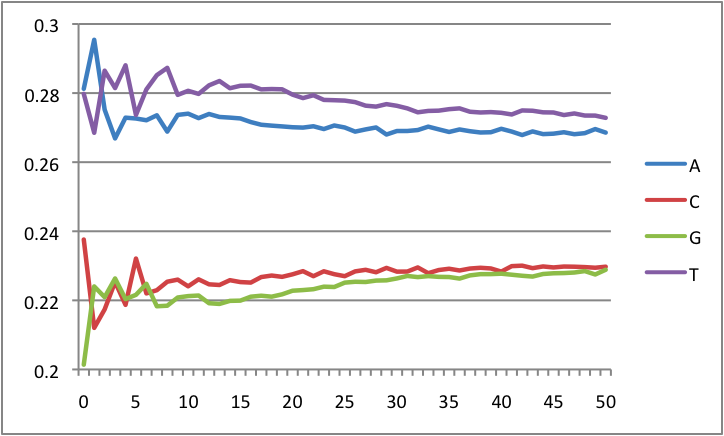

Supplement: Figure S2 — Basecalls for the read set D6 (NA12272) from the 1000 Genomes Project. X-axis showing the position in the read, y-axis the relative base frequency. (0.95 MB TIF) [file pone.0012681.s003.tif]

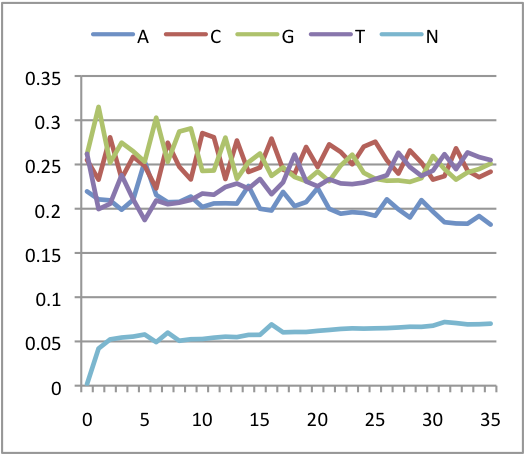

Supplement: Figure S3 — Basecalls for the read set D3 (NA11829) from the 1000 Genomes Project. X-axis showing the position in the read, y-axis the relative base frequency. (0.72 MB TIF) [file pone.0012681.s004.tif]

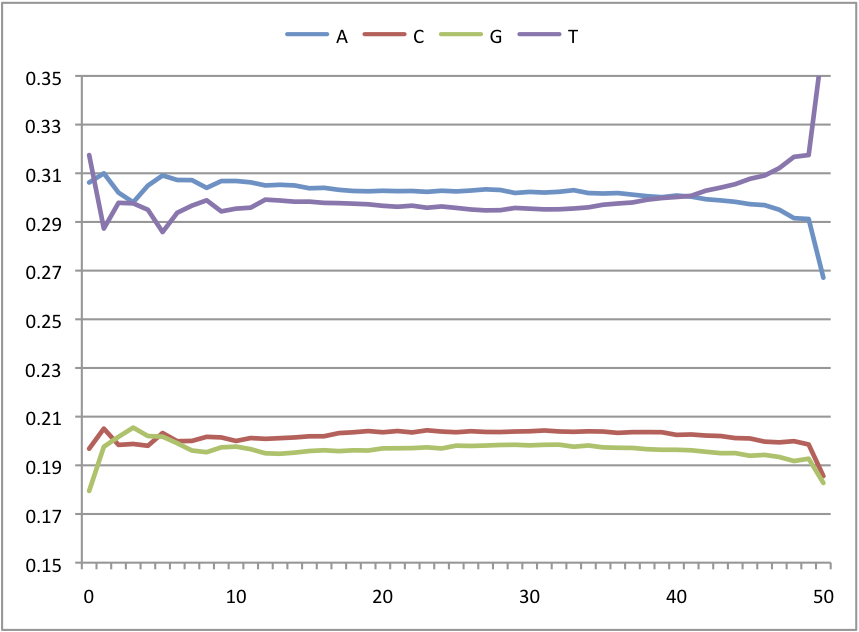

Supplement: Figure S4 — Basecalls for the read set D4 (NA12155) from the 1000 Genomes Project. X-axis showing the position in the read, y-axis the relative base frequency. (1.63 MB TIF) [file pone.0012681.s005.tif]

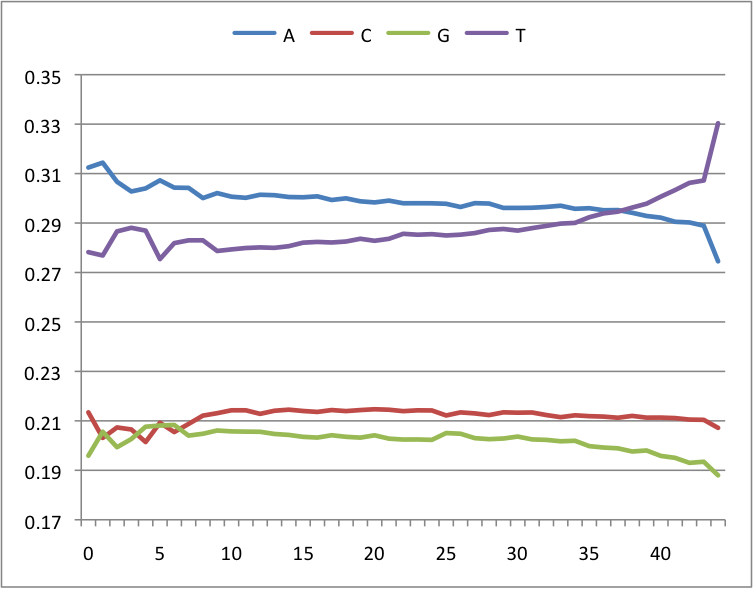

Supplement: Figure S5 — Basecalls for the read set D1 (SRX005986) from NCBI's Sequence Read Archive. X-axis showing the position in the read, y-axis the relative base frequency. (1.78 MB TIF) [file pone.0012681.s006.tif]

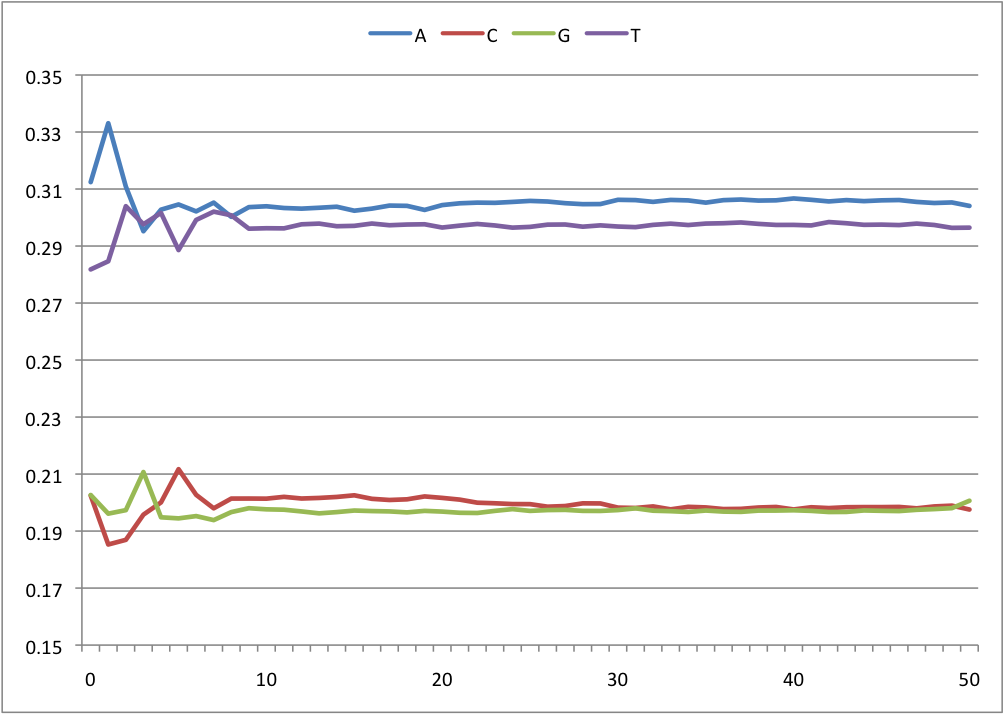

Supplement: Figure S6 — Basecalls for the read set D5 (NA10847) from the 1000 Genomes Project. X-axis showing the position in the read, y-axis the relative base frequency. (2.87 MB TIF) [file pone.0012681.s007.tif]

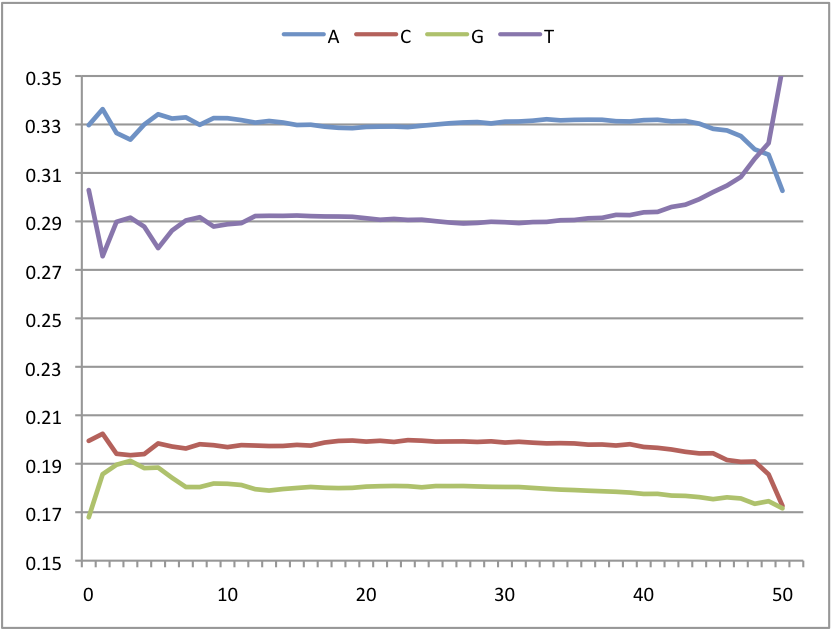

Supplement: Figure S7 — Basecalls for the read set D4* (NA12155) from the 1000 Genomes Project. X-axis showing the position in the read, y-axis the relative base frequency. (1.57 MB TIF) [file pone.0012681.s008.tif]

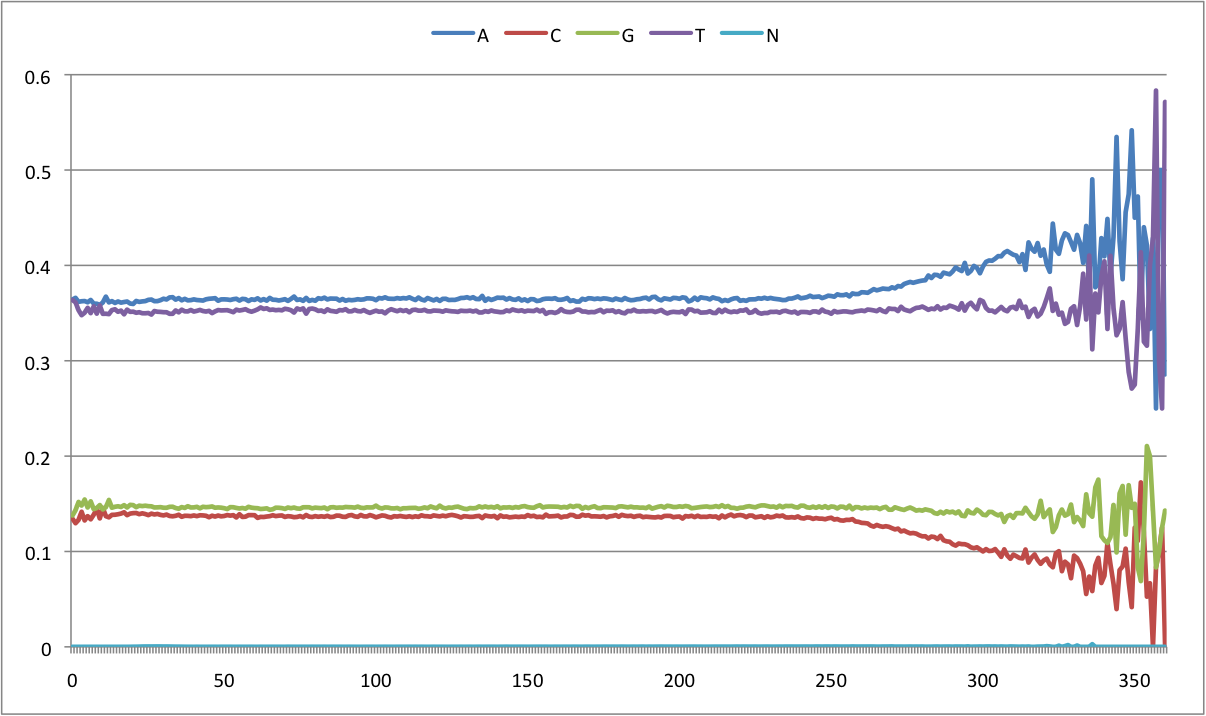

Supplement: Figure S8 — Basecalls for the read set D7 (SRX017210) from NCBI's Sequence Read Archive. X-axis showing the position in the read, y-axis the relative base frequency. Note that the graph is cut off at position 361, because only a very small number of reads exceeds this read length. (3.46 MB TIF) [file pone.0012681.s009.tif]

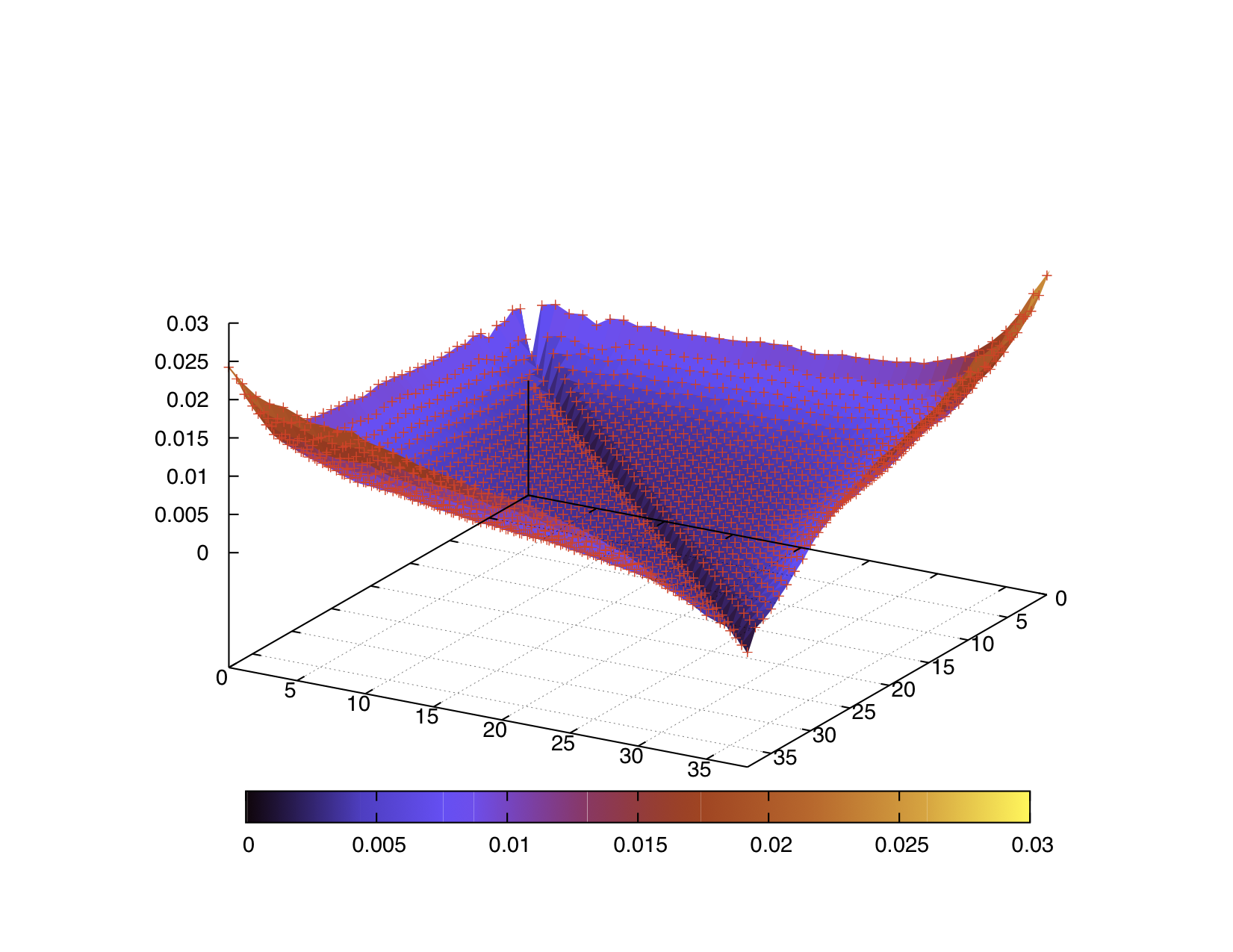

Supplement: Figure S9 — Kullback-Leiber divergence for the read set D1 (SRX005986) from NCBI's Short Read Archive. Data point represent KL(Pi∥Pj), x-axis indexing the first distribtion, y-axis the latter. Pi corresponds to the distribution of 6-mers at the ith position in a read. Note that the graph has been trimmed of the last position's distribution because of the high error rates. (6.32 MB TIF) [file pone.0012681.s010.tif]

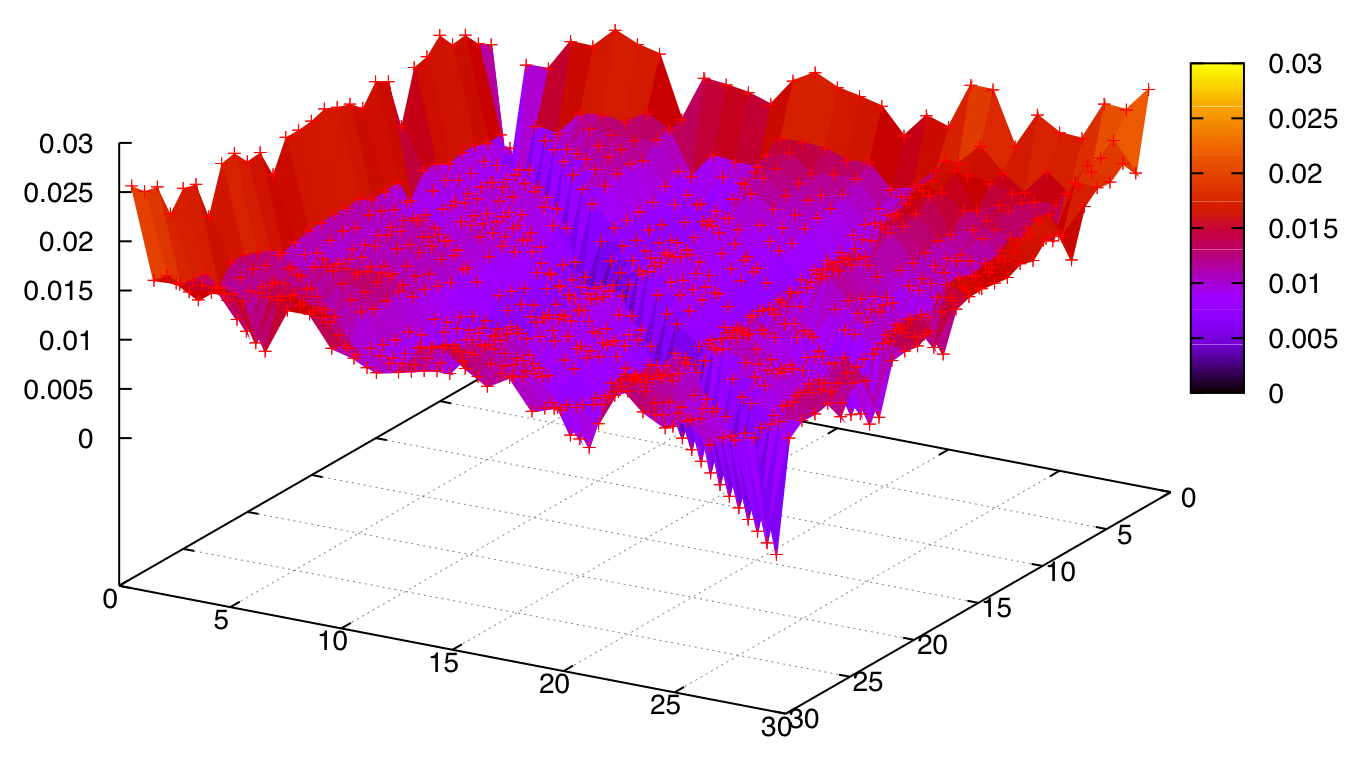

Supplement: Figure S10 — Kullback-Leiber divergence for the read set D2 (NA06985) from the 1000 Genomes Project. Data point represent KL(Pi∥Pj), x-axis indexing the first distribtion, y-axis the latter. Pi corresponds to the distribution of 6-mers at the ith position in a read. (3.12 MB TIF) [file pone.0012681.s011.tif]

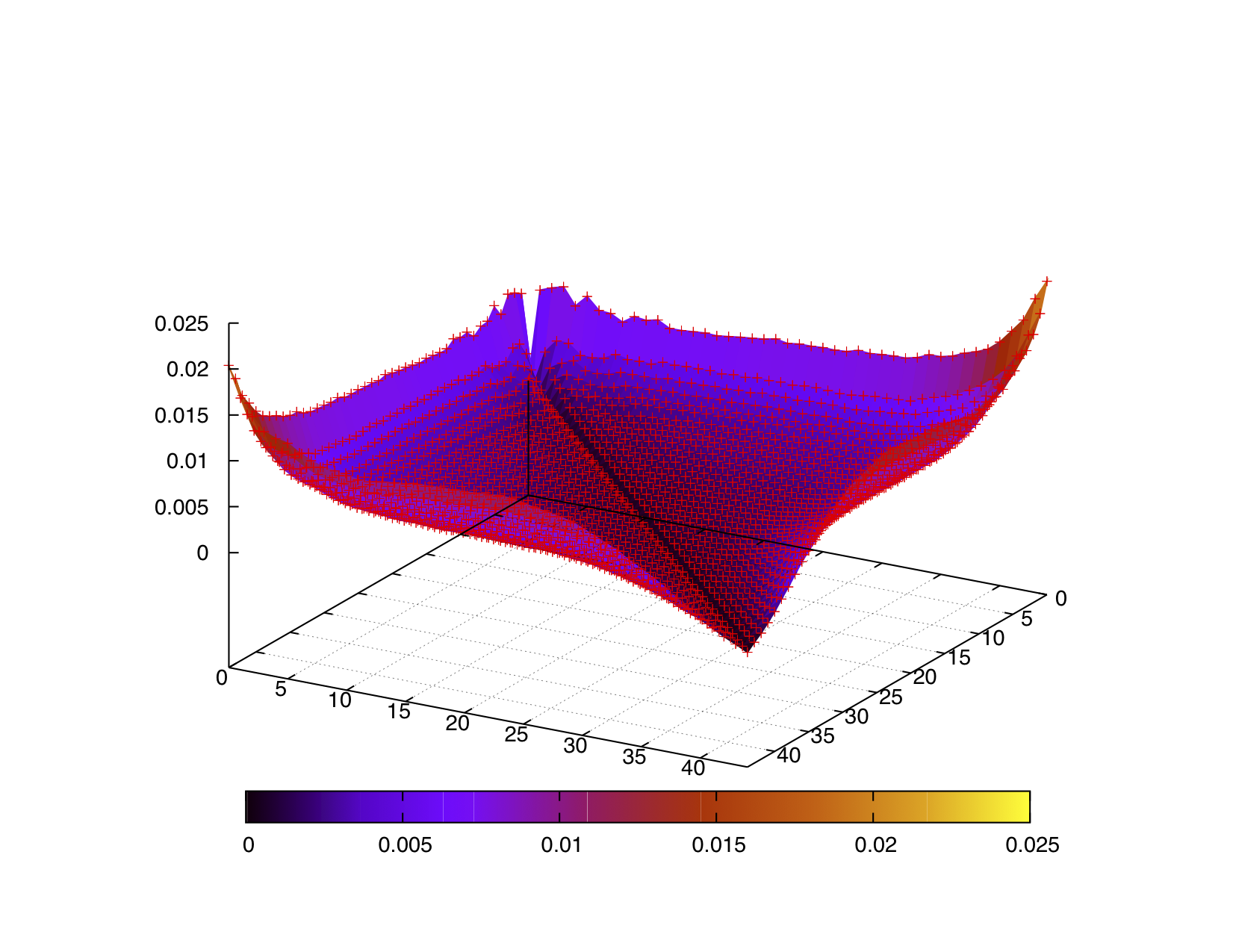

Supplement: Figure S11 — Kullback-Leiber divergence for the read set D4 (NA12155) from the 1000 Genomes Project. Data point represent KL(Pi∥Pj), x-axis indexing the first distribtion, y-axis the latter. Pi corresponds to the distribution of 6-mers at the ith position in a read. Note that the graph has been trimmed of the last position's distribution because of the high error rates. (6.32 MB TIF) [file pone.0012681.s012.tif]

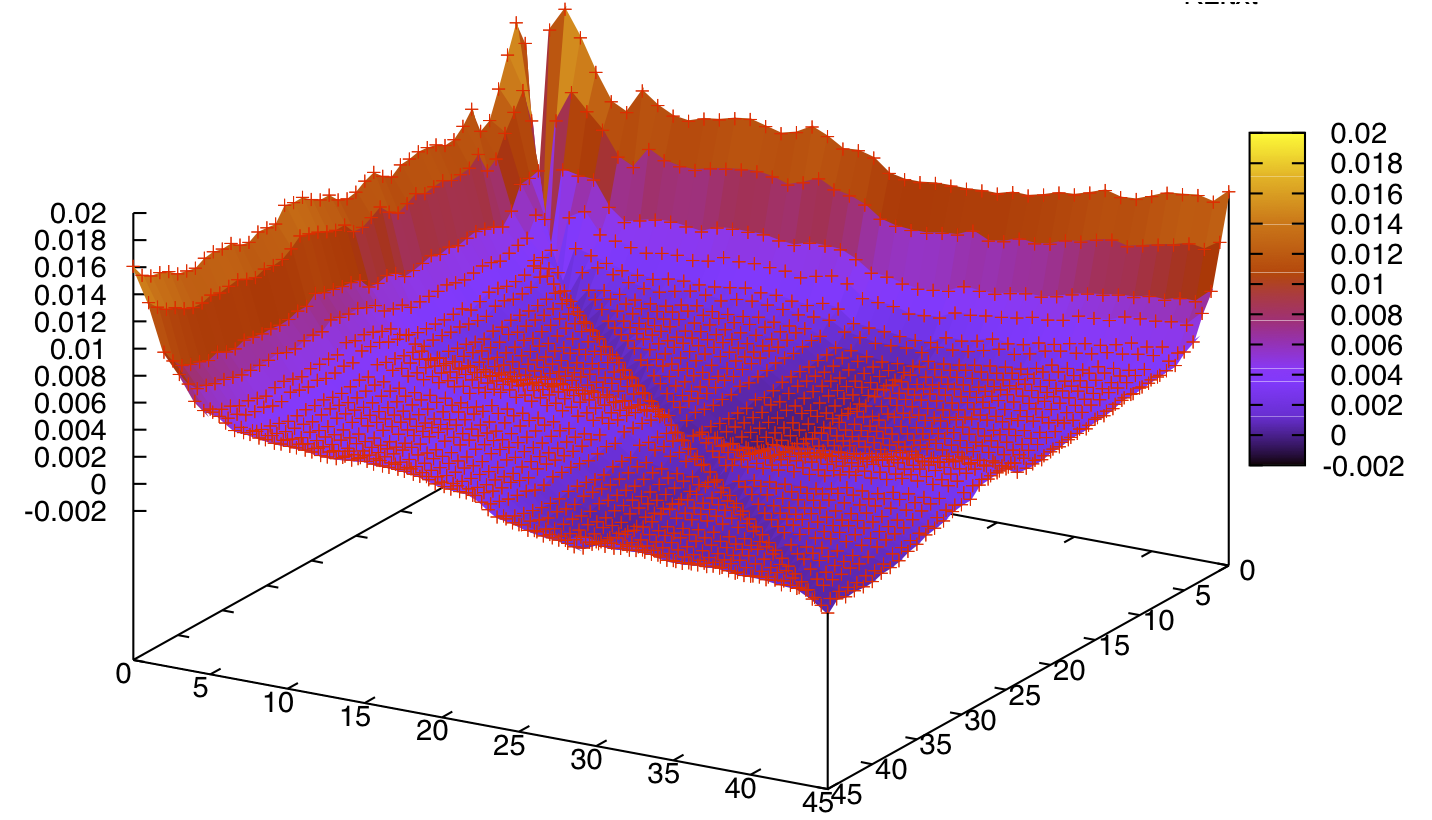

Supplement: Figure S12 — Kullback-Leiber divergence for the read set D5 (NA10847) from the 1000 Genomes Project. Data point represent KL(Pi∥Pj), x-axis indexing the first distribtion, y-axis the latter. Pi corresponds to the distribution of 6-mers at the ith position in a read. (3.52 MB TIF) [file pone.0012681.s013.tif]

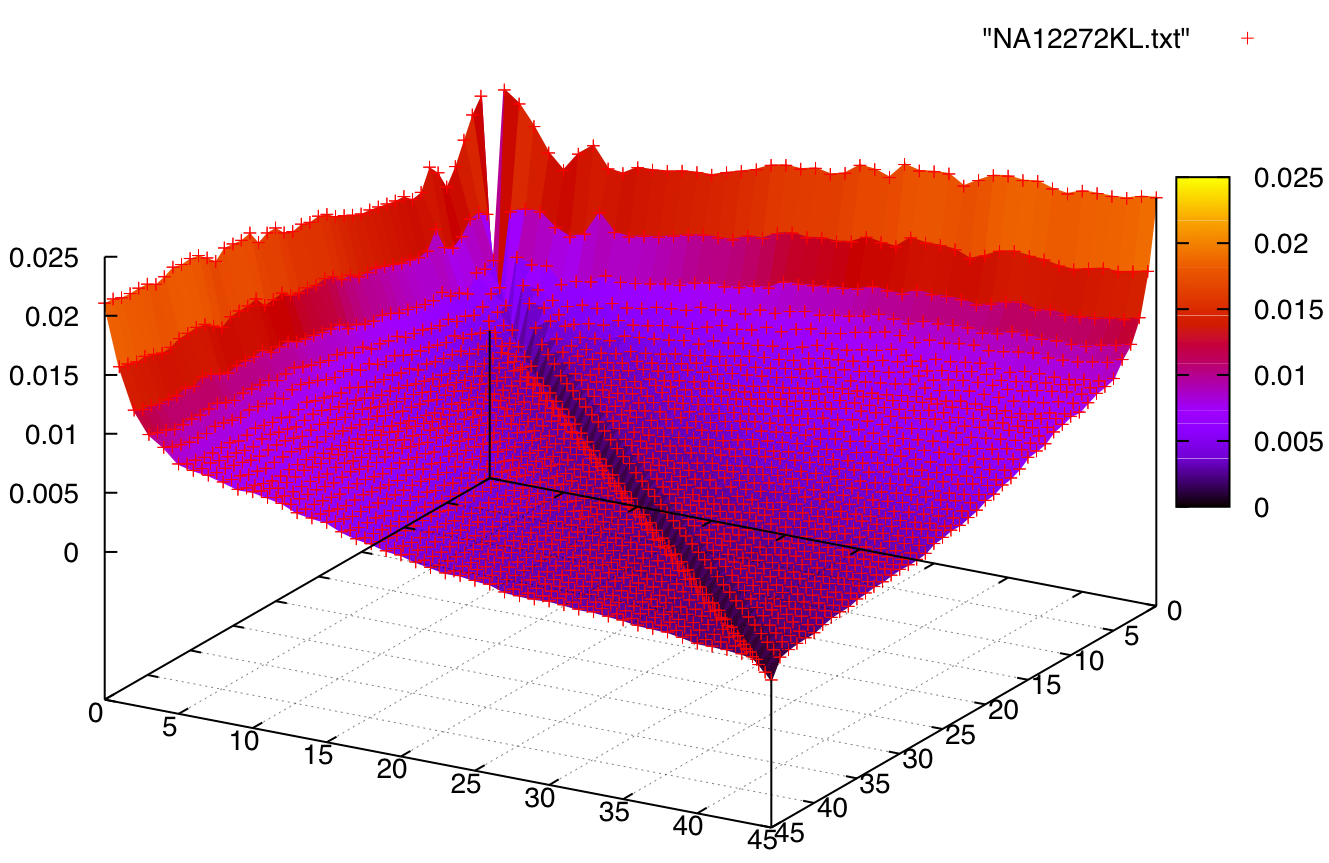

Supplement: Figure S13 — Kullback-Leiber divergence for the read set D6 (NA12272) from the 1000 Genomes Project. Data point represent KL(Pi∥Pj), x-axis indexing the first distribtion, y-axis the latter. Pi corresponds to the distribution of 6-mers at the ith position in a read. (3.44 MB TIF) [file pone.0012681.s014.tif]

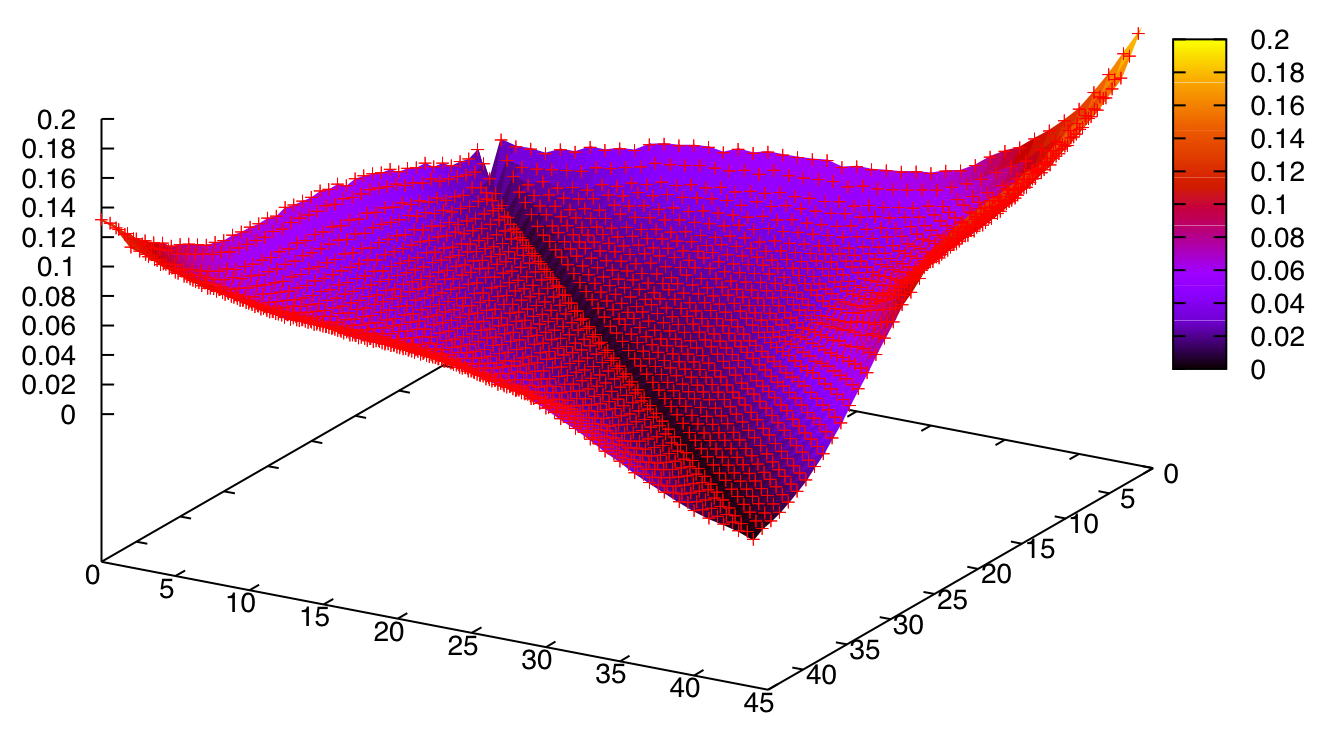

Supplement: Figure S14 — Kullback-Leiber divergence for a chip-seq data set. Data point represent KL(Pi∥Pj), x-axis indexing the first distribtion, y-axis the latter. Pi corresponds to the distribution of 6-mers at the ith position in a read. (2.91 MB TIF) [file pone.0012681.s015.tif]

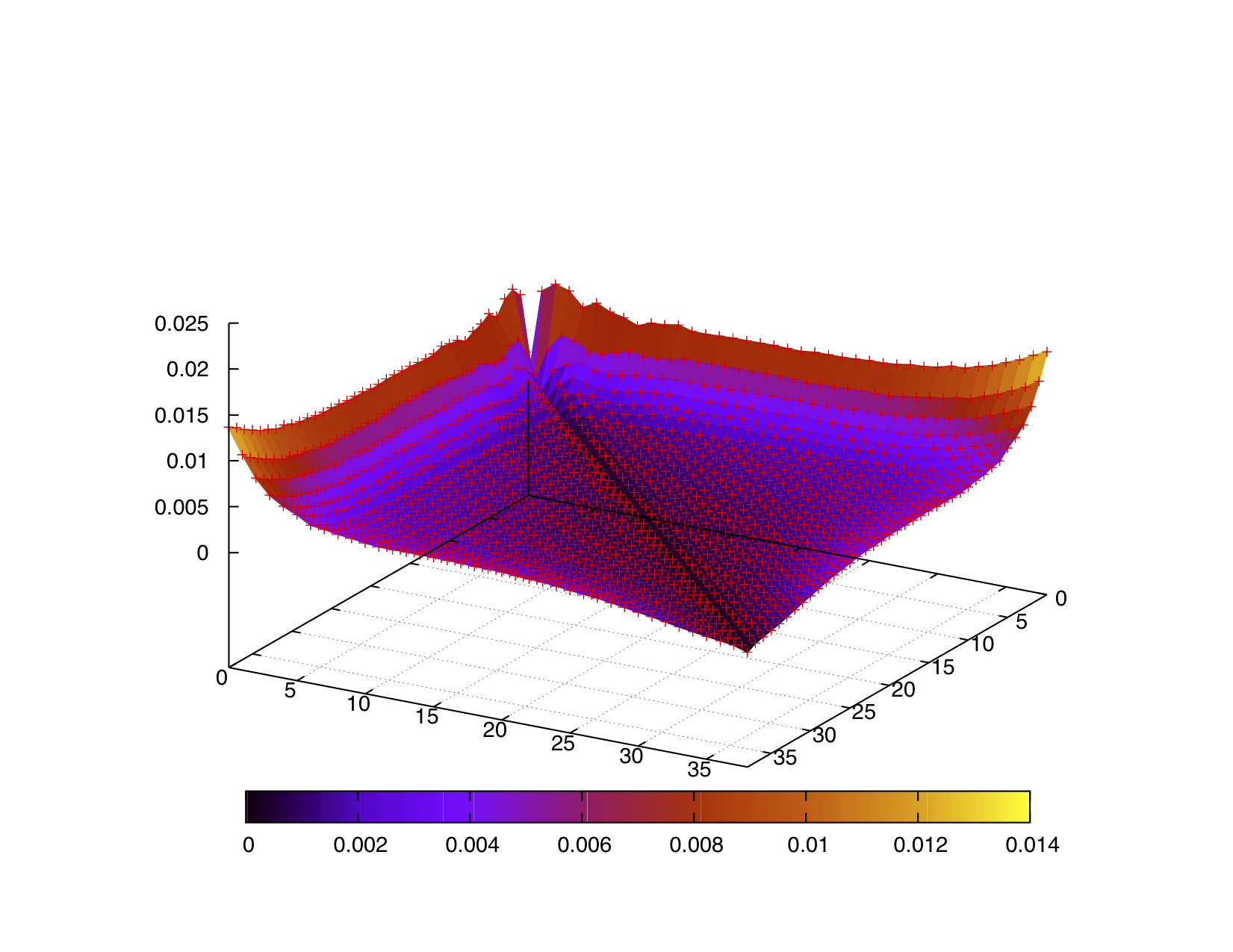

Supplement: Figure S15 — Kullback-Leiber divergence for the read set D4* (NA12155) from the 1000 Genomes Project. Data point represent KL(Pi∥Pj), x-axis indexing the first distribtion, y-axis the latter. Pi corresponds to the distribution of 6-mers at the ith position in a read. (6.32 MB TIF) [file pone.0012681.s016.tif]

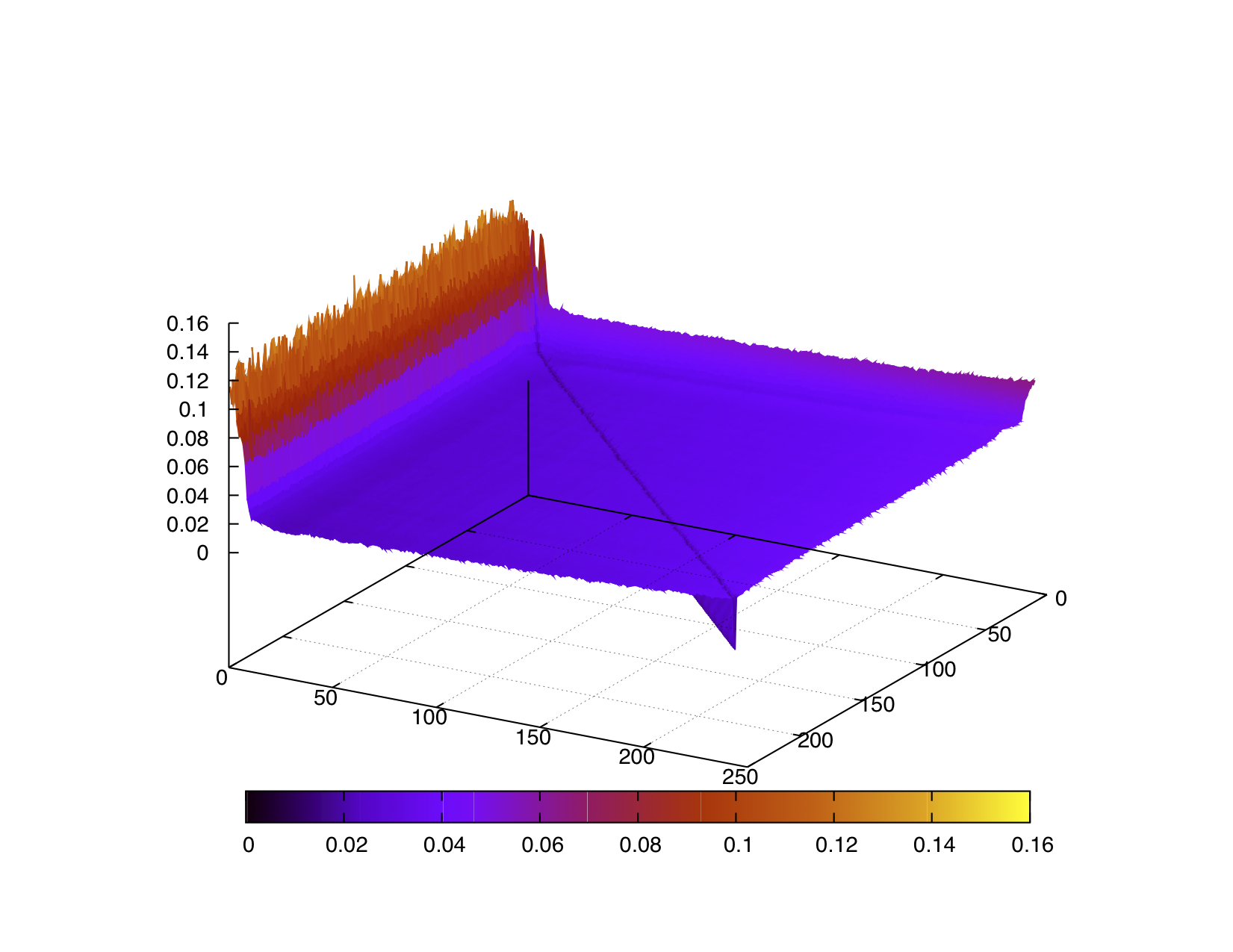

Supplement: Figure S16 — Kullback-Leiber divergence for the read set D7 (SRX017210) from NCBI's Short Read Archive. Data point represent KL(Pi∥Pj), x-axis indexing the first distribtion, y-axis the latter. Pi corresponds to the distribution of 6-mers at the ith position in a read. Note that the graph is only displayed up to postition 250, since the very low number of reads exceeding this read length makes comparison of distributions difficult and little meaningful. (6.32 MB TIF) [file pone.0012681.s017.tif]
